# A molecular quantitative trait locus map for osteoarthritis

**DOI:** 10.1038/s41467-021-21593-7

**Published:** 2021-02-26

**Authors:** Julia Steinberg, Lorraine Southam, Theodoros I. Roumeliotis, Matthew J. Clark, Raveen L. Jayasuriya, Diane Swift, Karan M. Shah, Natalie C. Butterfield, Roger A. Brooks, Andrew W. McCaskie, J. H. Duncan Bassett, Graham R. Williams, Jyoti S. Choudhary, J. Mark Wilkinson, Eleftheria Zeggini

**Affiliations:** 1 Institute of Translational Genomics, Helmholtz Zentrum München – German Research Center for Environmental Health, Neuherberg, Germany; 2 Cancer Research Division, Cancer Council NSW, Sydney, NSW Australia; 3 Wellcome Sanger Institute, Hinxton, United Kingdom; 4 School of Public Health, The University of Sydney, Sydney, NSW Australia; 5 The Institute of Cancer Research, London, United Kingdom; 6 Department of Oncology and Metabolism, University of Sheffield, Sheffield, United Kingdom; 7 Molecular Endocrinology Laboratory, Department of Metabolism, Digestion and Reproduction, Imperial College London, London, United Kingdom; 8 Division of Trauma & Orthopaedic Surgery, Department of Surgery, University of Cambridge, Cambridge, United Kingdom; 9 Centre for Integrated Research into Musculoskeletal Ageing and Sheffield Healthy Lifespan Institute, University of Sheffield, Sheffield, United Kingdom; 10 TUM School of Medicine, Technical University of Munich and Klinikum Rechts der Isar, Munich, Germany

**Keywords:** Gene expression profiling, Gene regulation, Genome-wide association studies, Transcriptomics

## Abstract

Osteoarthritis causes pain and functional disability for over 500 million people worldwide. To develop disease-stratifying tools and modifying therapies, we need a better understanding of the molecular basis of the disease in relevant tissue and cell types. Here, we study primary cartilage and synovium from 115 patients with osteoarthritis to construct a deep molecular signature map of the disease. By integrating genetics with transcriptomics and proteomics, we discover molecular trait loci in each tissue type and omics level, identify likely effector genes for osteoarthritis-associated genetic signals and highlight high-value targets for drug development and repurposing. These findings provide insights into disease aetiopathology, and offer translational opportunities in response to the global clinical challenge of osteoarthritis.

## Abstract (web summary)

Understanding the molecular effects of disease variants in relevant tissues is essential to understanding and treating disease. Here, the authors discover expression and protein quantitative trait loci in cartilage and synovium from 115 osteoarthritis patients to pinpoint genes of action and potential drug treatments.

## Introduction

Osteoarthritis is a severe, debilitating disease that affects the whole joint organ and is hallmarked by cartilage degeneration and synovial hypertrophy. As of 2019, osteoarthritis is estimated to affect 528 Million people worldwide and be the 15th leading cause of years lived with disability^1^. The lifetime risk of developing symptomatic knee and hip osteoarthritis is estimated to be 45 and 25%, respectively^2,3^, and is on an upward trajectory commensurate with rises in obesity and the ageing population. Between 2010 and 2019, the global prevalence of osteoarthritis and the resulting years lived with disability have both risen by 27.5%^1^. In 2013, osteoarthritis cost the United States $304 Billion^4^ and was the second most costly health condition treated at US hospitals, accounting for 4.3% of the combined costs for all hospitalisations^5^. Osteoarthritis-associated reduced physical activity results in a standardised all-cause mortality ratio of 1.55 (95% confidence interval 1.41 to 1.70) for its sufferers versus the general population^6^. Disease management focusses on alleviating pain, and in end-stage disease the only treatment is joint replacement surgery, emphasising the clear and urgent need to develop new therapies. To achieve this, we need to improve our understanding of the underlying molecular pathophysiology.

Epidemiological risk factors for the disease have been well-established and include older age, female sex, obesity, joint morphology and injury, and family history. The heritability of osteoarthritis has been estimated to range between 40 (for knee osteoarthritis) and 60% (for hip osteoarthritis)^7^. Genome-wide association studies (GWAS) have identified ~90 robustly-replicating risk loci^8^. However, the molecular landscape of osteoarthritis-relevant tissue has not been similarly characterised by large-scale efforts such as the GTEx^9^, ENCODE^10^ and RoadMap Epigenomics^11^ projects.

In this work, we perform a deep characterisation of the transcriptional and proteomic landscape of disease in chondrocytes and synoviocytes extracted from primary joint tissue of osteoarthritis patients. We define molecular quantitative trait loci, identify likely effector genes for GWAS signals, characterise molecular features of cartilage degradation and highlight drug development and repurposing opportunities through analysis of transcriptional signature changes.

## Results

### Generation of human joint tissue molecular profiles

**Fig. 1:**
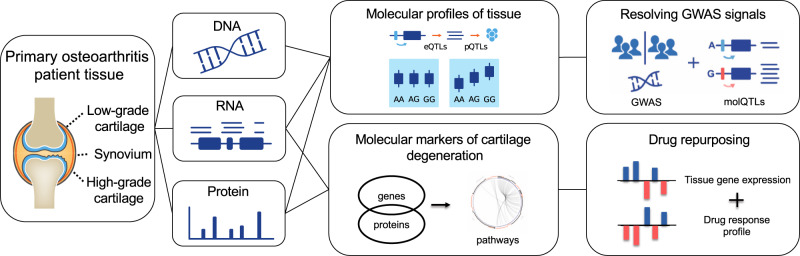
Study design leveraging multi-omics profiling from osteoarthritis patient tissues. We examined the molecular characteristics of osteoarthritis by profiling mRNA and proteins from low-grade cartilage, high-grade cartilage and synovium tissue of over 100 patients undergoing total joint replacement for osteoarthritis, and combining these data with patient genotypes. We identified genetic variants influencing mRNA or protein levels, several of which co-localise with genetic risk variants for osteoarthritis. We also identified molecular markers of cartilage degeneration, creating a gene expression profile of degeneration, and shortlisting existing drugs or compounds that reverse this profile in cell experiments.

We collected and characterised low-grade (intact; preserved) and high-grade (highly degraded; lesioned) cartilage, and synovial tissue from patients undergoing joint replacement for osteoarthritis (see Methods). The availability of paired low-grade and high-grade cartilage samples enables the comparison between these two disease states in affected primary tissue within the same individual, with high-grade cartilage showing more advanced cartilage degradation. All three tissues were profiled by RNA sequencing, and cartilage samples were additionally profiled by quantitative proteomics (Fig. 1 and Supplementary Fig. 1). We generated genome-wide genotype data from peripheral blood to define molecular quantitative trait loci (molQTLs) in each tissue type and omics level.

### Molecular QTLs in osteoarthritis tissues

**Fig. 2:**
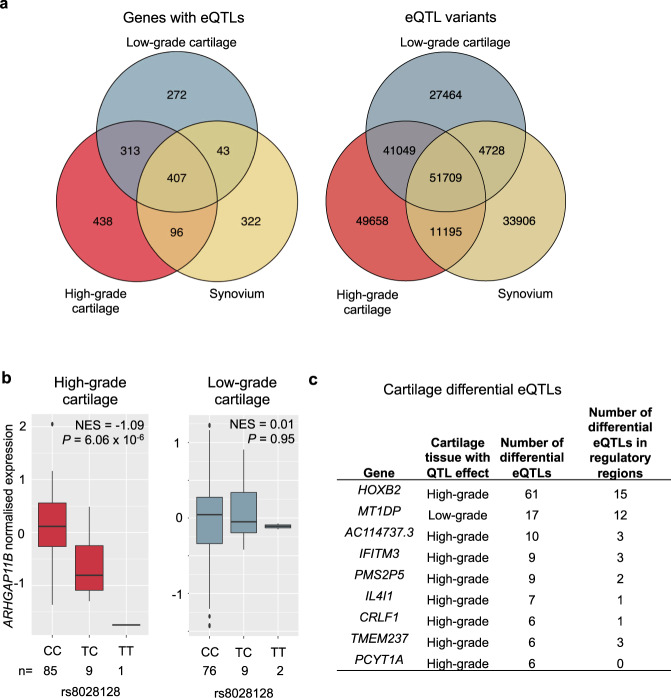
Molecular QTLs in osteoarthritis disease tissue. **a** eQTL overlap between tissues, for a total of 1891 genes with a least one eQTL (left) and 219,709 eQTL gene-variant pairs (right). 49% of detected eQTLs are not tissue-specific. **b** An example of differential QTL effect: a molecular QTL present in high-grade, but not low-grade cartilage (posterior probability *m* > 0.9 and *m* < 0.1, respectively), or vice versa. The boxplots show expression (residuals after regressing the normalised expression data on the 15 PEER factors, sex and array) at 25th to 75th percentiles, with centre at the median and whiskers extend to 1.5 times the interquartile range. NES FastQTL normalised effect size, *P* FastQTL association *P* value, n number of individuals included in the analysis for each genotype. **c** Genes with ≥5 differential eQTL variants (all genes see Supplementary Data 1).

Identification of molecular QTLs can provide a better understanding of the transcriptional regulation of key cell types across disease stages. We identified *cis* expression QTLs (*cis*-eQTLs) for 1891 genes in at least one tissue (Fig. 2), with high correlation of effects across the tissues studied (Supplementary Fig. 2). The direction of effect was concordant across all *cis-*eQTLs detected in both low-grade and high-grade cartilage. We identified *cis* protein QTLs (*cis*-pQTLs) for 38 genes in at least one tissue, with a similarly strong correlation across low-grade and high-grade cartilage (Supplementary Figs. 3, 4 and Supplementary Note 1).

### Differential genetic regulation of gene expression

We identified differential regulation of gene expression between high-grade and low-grade cartilage, with 172 variants showing strong evidence for an eQTL effect in one tissue grade (posterior probability >0.9), but not in the other (posterior probability <0.1), termed ‘differential eQTLs' (Fig. 2, Supplementary Figs. 5, 6, and Supplementary Data 1). We detected 32 genes with differential eQTLs (Supplementary Data 1). These genes function in the regulation of gene expression (high-grade eQTL: *HOXB2, IFITM3, EIF2B3TRAF2, HLCS, APBA1, HLX*; low-grade eQTL: *EARS2, TCEB1, USP16*), nervous system development (high-grade eQTL: *HOXB2, CRLF1, EIF2B3, APBA1, HLX, NEGR1, ARGHAP11B*; low-grade eQTL: *SZT2, NRN1*), response to stress (high-grade eQTL: *IFITM3, EIF2B3, TRAF2, ICAM3*; low-grade eQTL: *PNKP, SZT2, REV1, USP16*), immune response (high-grade eQTL: *IFITM3, IL4I1, CRLF1, TRAF2, ICAM3, HLX*), cell adhesion (high-grade eQTL: *TRAF2, ICAM3, APBA1, HLX, NEGR1*) and catabolic processes (high-grade eQTL: *IL4I1, TRAF2, WDR91, HAAO*; low-grade eQTL: *USP16*). For most of these processes, some genes with differential eQTLs show gain of genetic regulatory associations in high-grade cartilage, while others show loss of such associations compared to low-grade cartilage, suggesting a broader rewiring of regulatory processes (see Supplementary Note 2). Sixteen genes have differential eQTLs located in a regulatory region.

### Co-localisation of GWAS signals and molecular QTLs in disease tissue

**Fig. 3:**
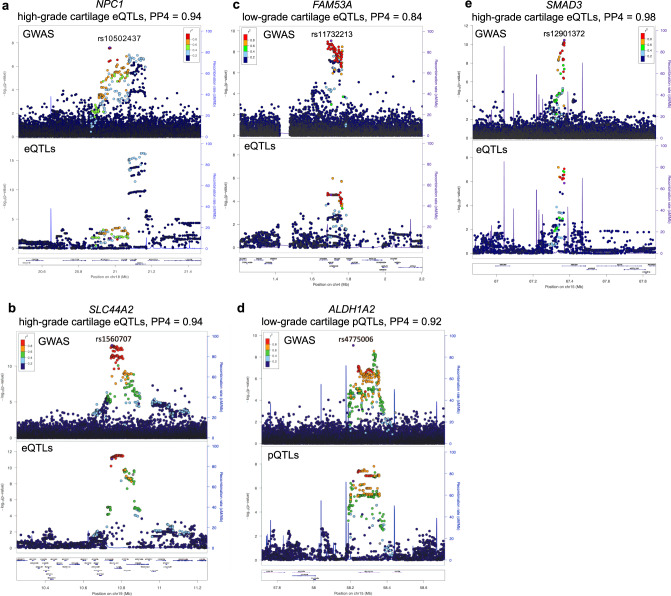
GWAS and molecular QTL *P* values in regions with co-localisation of the associations. GWAS and molecular QTL *P* values in regions with co-localisation of the associations. PP4 posterior probability for co-localisation. **a** *NCP1* eQTLs in high-grade cartilage (**b**) *SLC44A2* eQTLs in high-grade cartilage (**c**) *FAM53A* eQTLs in low-grade cartilage (**d**) *ALDH1A2* pQTLs in low-grade cartilage (**e**) *SMAD3* eQTLs in high-grade cartilage. For *NPC1* and *SMAD3*, Supplementary Fig. 7 shows co-localisation with low-grade cartilage molecular QTLs.

Having established these cell- and disease stage-specific maps of molecular QTLs, we used them to identify effector genes driving GWAS signals, the majority of which reside in non-coding sequence. Co-localisation analysis can indicate whether the same variant underpins association with both disease and gene expression levels. We found strong evidence for co-localisation of five osteoarthritis signals with cartilage molQTLs for *ALDH1A2*, *NPC1*, *SMAD3*, *FAM53A* and *SLC44A2* (Table 1, Fig. 3, and Supplementary Fig. 7). In all five instances, the GWAS index variant is non-coding. In three cases (*ALDH1A2, SMAD3* and *SLC44A2*), the likely effector gene is the one closest to the lead variant. For the *NCP1* and *FAM53A* loci, the lead variants reside in introns of the *TMEM241* and *SLBP* genes, 141 and 18 kb away from the likely effector gene, respectively.

**Table 1:** Osteoarthritis GWAS signals with high posterior probability for co-localisation with molecular QTLs.

GWAS variant^a^	rs10502437	rs11732213	rs12901372	rs1560707	rs4775006
Osteoarthritis phenotype	All	Hip/Knee	Hip	All	Knee
Risk allele frequency	0.6	0.81	0.53	0.37	0.41
Odds ratio	1.03	1.06	1.08	1.04	1.06
*P* value	2.50 × 10^–8^	8.81 × 10^–10^	3.46 × 10^–11^	1.35 × 10^–13^	8.40 × 10^–10^
Risk allele	A	T	C	T	A
Gene	*NPC1*	*FAM53A*	*SMAD3*	*SLC44A2*	*ALDH1A2*
Risk allele effect on gene expression	decrease	decrease	decrease	increase	increase

^a^Signals are denoted by their lead variants.

### Molecular hallmarks of cartilage degradation

To identify molecular signatures associated with disease severity, we characterised gene expression and protein abundance differences between high-grade and low-grade cartilage in the largest sample set to date. We detected significant expression differences for 2557 genes and abundance differences for 2233 proteins at 5% false discovery rate (FDR) (Supplementary Figs. 8, 9, and Supplementary Notes 3, 4). A total of 409 genes (Supplementary Data 2) demonstrated significant differential expression at both the RNA and protein levels, lending robust cross-omics evidence for their involvement in disease progression. In keeping with previous smaller-scale reports^12–14^, extracellular matrix (ECM)-receptor interaction was the primarily activated pathway in high-grade compared to low-grade cartilage (Supplementary Note 5, Supplementary Fig. 10, and Supplementary Data 3).

We used an independent, previously published analysis of RNA sequencing data from 35 osteoarthritis patients^15^ to replicate the observed molecular differences between high-grade and low-grade cartilage (within the power constraints of the smaller replication set). Of the differentially expressed genes and proteins, 65.9 and 68.3% showed a concordant direction of effect in the replication data, respectively (both Fisher’s *p* < 10^−10^, Supplementary Data 4). This concordance increased to 77.9% for genes with cross-omics concordant differential expression in this study, indicating additional robustness afforded by cross-omics data integration. We found significantly higher concordance where replication power was highest (88.5% for genes with cross-omics higher expression in high-grade cartilage, compared to 66.7% for genes with cross-omics lower expression in high-grade cartilage, Fisher’s *p* = 8.6 × 10^−6^, Supplementary Data 4).

### Differentially expressed genes with genetic associations

Ninety-one of the genes with significantly different expression profiles between high- and low-grade cartilage were also associated with genetic risk of osteoarthritis (i.e. among the 238 genes with gene-level significant association in a recent meta-analysis^8^; Supplementary Data 5). A total of 54 genes also showed concordant molecular differences in the independent replication dataset, providing further evidence for the potential involvement of these genes in osteoarthritis disease processes. For example, variants in *ALDH1A2* are associated with knee osteoarthritis, and we found significantly higher *ALDH1A2* gene expression and lower protein abundance in high-grade cartilage (suggesting a potential role for post-transcriptional regulation for this gene). For *SLC39A8*, the GWAS signal was fine-mapped with posterior probability of 0.999 to a single missense variant predicted to be possibly deleterious by PolyPhen-2^16^, and the gene demonstrated higher expression levels in high-grade cartilage.

### Candidate therapeutic compounds and drug targets

Having characterised the molecular differences between low-grade and high-grade cartilage in osteoarthritis, we sought to identify compounds capable of reversing these changes.

Using in vitro drug screen data from ConnectivityMap^17^, we identified 19 compounds that induced strong opposing gene expression signatures, reducing the expression of genes with cross-omics higher expression in high-grade cartilage (Table 2, Supplementary Data 6, and Supplementary Note 6). Several of these compounds foster known biological relevance to osteoarthritis, including the oestrogen receptor agonists diethylstilbestrol and alpha-estradiol, consistent with established epidemiological data showing an association between osteoarthritis and oestrogen deficiency^18^. Although studies of oestrogen therapy for osteoarthritis have been inconclusive^19,20^, this screening approach could facilitate further refinement of existing drug groups and allow more focussed investigational molecule development.

**Table 2:** Compounds with strongest evidence for inducing gene expression signatures that counter differences between high-grade and low-grade cartilage, based on data from ConnectivityMap^17^ and the clue.io platform.

Name^a^	Description	DE targets^b^
Emetine	protein synthesis inhibitor	RPS2 (P+)
Rucaparib	PARP inhibitor	PARP2 (R−)
Alpha-estradiol	oestrogen receptor agonist	KCNMA1 (R−, P−)
VEGF-receptor-2-kinase-inhibitor-IV	VEGFR inhibitor	
IB-MECA	adenosine receptor agonist, granulocyte colony stimulating factor agonist	
Diethylstilbestrol	oestrogen receptor agonist, chloride channel blocker	
KIN001-220	Aurora kinase inhibitor	
SB-216763	glycogen synthase kinase inhibitor	GSK3B (R+, P+), CDK2 (P−)
RHO-kinase-inhibitor-III[rockout]	ROCK inhibitor	IMPDH2 (P−)
Nornicotine	acetylcholine receptor agonist	

^a^The 10 compounds with strongest evidence based on median of cell lines are shown; full results see Supplementary Data 6.

^b^Drug targets as listed in ConnectivityMap with differential expression between high-grade and low-grade cartilage on RNA (R) or protein (P) level, ‘+' and ‘−' indicate higher or lower expression high-grade cartilage, respectively.

Further notable examples of compounds with the potential to reverse molecular changes include IB-MECA, VEGF-receptor-2-kinase-inhibitor-IV and nornicotine. IB-MECA is used as an anti-inflammatory drug in rheumatoid arthritis, and has been shown to prevent cartilage damage, osteoclast/osteophyte formation and bone destruction in a rat model of chemically-induced osteoarthritis^21^. VEGF-receptor-2-kinase-inhibitor-IV is a *VEGF* receptor inhibitor. *VEGF* modulates chondrocyte survival during development, is essential for bone formation and skeletal growth, and dysregulated in osteoarthritis^22^. A VEGFR2 kinase inhibitor has demonstrated therapeutic potential in mice^23^. Nornicotine is a demethylated derivative of nicotine found in plants, and there is a well-established epidemiological inverse relationship between smoking and osteoarthritis^8,24^. Thus, synthetic nicotine derivatives may have a candidate role in osteoarthritis prevention.

We further identified 36 genes for which knock-down or overexpression has the potential to reduce the molecular differences between high-grade and low-grade cartilage (Supplementary Data 6), for example knock-down of *IL11*. IL11 is a cytokine with a key role in inflammation, and several therapeutics that inhibit IL11 signalling are in development against a range of inflammatory and fibrotic diseases^25,26^. Variation in *IL11* is associated with increased risk of hip osteoarthritis^8^, and the gene is upregulated in osteoarthritis knee tissue^27^, showing the most significant upregulation in high-grade cartilage in the independent replication dataset^15^ (22.8-fold higher expression, FDR = 1.5 × 10^−20^). These findings provide strong supportive evidence for downregulation of *IL11* as a potential therapeutic intervention for osteoarthritis.

## Discussion

To date, deep molecular profiling of cells of relevance to osteoarthritis has been absent from large-scale efforts such as GTEx, ENCODE and RoadMap, in part because of the challenges associated with low cellularity and high ECM content of cartilage^28,29^. Here, we address this gap by the systematic study of paired low-grade and high-grade cartilage and synovium tissues from 115 patients with osteoarthritis to provide the first deep molecular QTL map of cell types directly involved in disease. These data are made publically available (see Data availability). We identify genotype-dependent, divergent patterns of gene regulation between diseased and healthy cartilage, and between cartilage and synovium, that underline the biological specificity of disease stage and cell type when investigating regulatory variant function.

The co-localisation of GWAS signals and molecular QTLs in disease tissue helps pinpoint the identity of causal genes for hithert unsolved association signals. A previous study^8^ examined the co-localisation of osteoarthritis GWAS signals with eQTLs from the GTEx resource^9^, which does not include cartilage. Several osteoarthritis GWAS signals were found to co-localise with eQTLs in only 1 or 2 of 53 tissues (e.g. *ALDH1A2*: ovary and tibial artery, *SMAD3*: skeletal muscle; *SLC44A2*: adrenal gland), without clear transferability of results to disease-relevant tissue. Here, we provide robust evidence that these three GWAS signals co-localise with molecular QTLs in primary cartilage. This availability of molQTL data from chondrocytes and synoviocytes offers a resource that will enable the resolution of further genetic association signals emerging from ongoing large-scale efforts in osteoarthritis (e.g. https://www.genetics-osteoarthritis.com) and further traits of musculoskeletal relevance, in which these cell types are of importance.

The strong correlation between RNA and protein level signatures within cell type and disease state and the contrast between disease states provides both strong internal consistency and with previous observations of ECM remodelling by chondrocytes in an inflammatory environment as a key modifiable molecular signature in the degeneration process^30,31^. We further demonstrate the external validity of these hallmark signatures by showing good concordance with publically-available RNA-seq data from a smaller, independent, human osteoarthritis cohort^15^, particularly for genes whose expression is increased in high-grade cartilage.

On a related theme, our identification of an association between 91 established osteoarthritis variants and disease state, cell-specific gene expression and protein profiles highlights the value of integrating multi-omics data with genetic association summary statistics to identify likely effector genes for GWAS signals, and hence targets for development of new therapies. The subsequent ConnectivityMap analyses built upon this theme to identify molecules with potential clinical application to reverse the molecular signatures characteristic of diseased chondrocytes, an in-silico complementary approach to conventional compound library screening. Here, we identify 19 molecules and 36 genes with biological supportive evidence for validation as candidate drugs in experimental models, such as that recently applied by Shi et al. to demonstrate that the small molecule BNTA stimulates expression of ECM components while supressing inflammatory mediators in human osteoarthritic cartilage, an effect mediated through superoxide dismutase 3^30^.

Although these data demonstrate the context-specificity of molecular indicators of disease, this work also has limitations. Cartilage remains a difficult tissue to study at the molecular level, given the low cell accessibility. The analyses were performed on extracted tissues characterised by macroscopic grading using the International Cartilage Repair Society (ICRS) scoring system^32^ and although all tissues were collected from weight-bearing areas (i.e. mechanically-loaded), a degree of cell disease state heterogeneity is inevitable in the pooled sample collection. However, the collection of both disease states from the same individual eliminates inter-individual variation as a source of confounding. Finally, although these data represent the largest cohort of its depth and kind in osteoarthritis, they are observational in nature. Future biological experiments (e.g. by gene knock-out or over-expression) will be key to prove which gene expression changes are causal in disease development and progression. The genes with convergent evidence from this study as well as osteoarthritis GWAS are presented to help such research and accelerate the pathway to translation.

In summary, by integrating multiple layers of omics data, we have generated a first molecular QTL map for osteoarthritis-relevant tissues and have helped resolve genetic association signals by identifying likely effector genes. We demonstrate how integrating multi-omics data in primary human complex disease tissue can serve as a valuable approach that moves from basic discovery to accelerated translational opportunities. Our findings identify drug repurposing opportunities and allow novel investigational avenues for therapy development, responding to the urgent clinical need of patients suffering from osteoarthritis.

## Methods

### Study participants

We collected tissue samples from 115 patients undergoing total joint replacement surgery in 4 cohorts: 12 knee osteoarthritis patients (cohort 1; 2 women, 10 men, age 50–88 years, mean 68 years); 20 knee osteoarthritis patients (cohort 2; 14 women, 6 men, age 54–82 years, mean 70 years); 13 hip osteoarthritis patients (cohort 3; 8 women, 5 men, age 44–84 years, mean 62 years); 70 knee osteoarthritis patients (cohort 4; 42 women, 28 men, age 38–84 years, mean 70 years).

All patients provided written, informed consent prior to participation in the study. Matched low-grade and high-grade cartilage samples were collected from each patient, while synovial lining samples were collected from patients in cohorts 2 and 4. All cartilage samples were collected from weight-bearing areas of the joint to ensure that any differences observed between low- and high-grade cartilage reflect disease progression stage rather than differential biomechanical stress.

#### Cohorts 1, 2, 4 (knee osteoarthritis)

This work was approved by Oxford NHS REC C (10/H0606/20 and 15/SC/0132), and samples were collected under Human Tissue Authority license 12182, Sheffield Musculoskeletal Biobank, University of Sheffield, UK.

We confirmed a joint replacement for osteoarthritis, with no history of significant knee surgery (apart from meniscectomy), knee infection, or fracture, and no malignancy within the previous 5 years. We further confirmed that no patient used glucocorticoid use (systemic or intra-articular) within the previous 6 months, or any other drug associated with immune modulation. For cohort 1, cartilage samples were scored using the OARSI cartilage classification system^33,34^. From each patient, we obtained one sample with high OARSI grade signifying high-grade degeneration ('high-grade sample'), and one cartilage sample with low OARSI grade signifying healthy tissue or low-grade degeneration ('low-grade sample').

For cohorts 2 and 4, cartilage samples were scored macroscopically using the ICRS scoring system^32^. From each patient, we obtained one sample of ICRS grade 3 or 4 signifying high-grade degeneration ('high-grade sample'), and one cartilage sample of ICRS grade 0 or 1 signifying healthy tissue or low-grade degeneration ('low-grade sample'). For cohorts 2 and 4, we also collected synovial membrane from the suprapatellar region of the knee joint.

Finally, from all patients in cohorts 1, 2 and 4, we also obtained a blood sample to extract DNA for genotyping.

#### Cohort 3 (hip osteoarthritis)

Samples were collected under National Research Ethics approval reference 11/EE/0011, Cambridge Biomedical Research Centre Human Research Tissue Bank, Cambridge University Hospitals, UK.

We confirmed osteoarthritis disease status by examination of the excised femoral head. From each patient, we obtained a cartilage sample showing a fibrillated or fissured surface signifying high-grade degeneration ('high-grade sample'), one cartilage sample showing a smooth shiny appearance signifying healthy tissue or low-grade degeneration ('low-grade sample').

### Isolation of chondrocytes

For cohorts 1, 2 and 4, we followed a previously established protocol to isolate chondrocytes^12^ with the details as follows. Osteochondral samples were transported in Dulbecco’s modified Eagle’s medium (DMEM)/F-12 (1:1) (Life Technologies) supplemented with 2 mM glutamine (Life Technologies), 100 U/ml penicillin, 100 μg/ml streptomycin (Life Technologies), 2.5 μg/ml amphotericin B (Sigma-Aldrich) and 50 μg/ml ascorbic acid (Sigma-Aldrich) (serum free media). Half of each sample was then taken forward for chondrocyte extraction. Cartilage was removed from the bone, dissected and washed twice in 1xPBS. Tissue was digested in 3 mg/ml collagenase type I (Sigma-Aldrich) in serum free media overnight at 37 °C on a flatbed shaker. The resulting cell suspension was passed through a 70 μm cell strainer (Fisher Scientific) and centrifuged at 400×*g* for 10 min. Subsequently, the cell pellet was washed twice in serum free media and centrifuged at 400×*g* for 10 min. The resulting cell pellet was resuspended in serum free media. Cells were counted using a haemocytometer and the viability checked using trypan blue exclusion (Invitrogen). The optimal cell number for spin column extraction from cells was between 4 × 10^6^ and 1 × 10^7^. Cells were then pelleted and homogenised.

For cohort 3, the extraction of chondrocytes has previously been described^35^ in the majority of these samples, with the remaining samples following the same protocol. The protocol was based on that for cohorts 1, 2, 4 and highly similar as described in the following. Each cartilage portion was minced with a scalpel and placed in 20 ml of Dulbecco’s modified Eagle medium (Invitrogen) containing 10% foetal bovine serum (Invitrogen) and 6 mgml^−1^ collagenase A (Sigma). The tissue culture flasks were incubated overnight to digest the cartilage pieces. The resulting cell suspension was passed through a 30 μm filter (Miltenyi) and centrifuged at 400×*g* for 10 min. The cell pellet was then re-suspended in 1 ml of PBS and counted on a haemocytometer following 1:1 mixing with trypan blue to determine cell viability.

### Isolation of synoviocytes

We followed a previously established protocol to process synovial samples^36^, with details as follows. Synovial samples were transported in serum free media, as described above. The synovial membrane was dissected from underlying tissue then trypsinised for 1 h. Tissue was then digested in 1 mg/ml Collagenase Blend H (Sigma Aldrich) in serum free media overnight at 37 °C on a flatbed shaker. The resulting cell suspension was passed through a 100 μm cell strainer (Fisher Scientific) and centrifuged at 400×*g* for 10 min. Subsequently, the cell pellet was washed twice in serum free media and centrifuged at 400×*g* for 10 min. The resulting cell pellet was resuspended in serum free media. Cells were counted using a haemocytometer and the viability checked using trypan blue exclusion (Invitrogen). The optimal cell number for spin column extraction from cells was between 4 × 10^6^ and 1 × 10^7^. Cells were then pelleted and homogenised.

### DNA, RNA and protein extraction

DNA, RNA, and protein extraction was carried out using Qiagen AllPrep DNA/RNA/Protein Mini Kit following the manufacturer’s instructions for cohorts 1, 2 and 4, with small variations for cohort 3 as previously described^35^ and recapitulated in the Supplementary Methods. Samples were frozen at −80 °C (cohorts 1, 2, 4) or −70 °C (cohort 3) prior to assays.

### RNA sequencing

We performed a gene expression analysis on samples from 113 patients (Supplementary Data 7). We purified poly-A tailed RNA (mRNA) from total RNA using Illumina’s TruSeq RNA Sample Prep v2 kits. We then fragmented the mRNA using metal ion-catalysed hydrolysis and synthesised a random-primed cDNA library. The resulting double-strand cDNA was used as the input to a standard Illumina library prep, whereby ends were repaired to produce blunt ends by a combination of fill-in reactions and exonuclease activity. We performed A-tailing to allow samples to be pooled, by adding an ‘A' base to the blunt ends and ligation to Illumina Paired-end Sequencing adaptors containing unique index sequences. Due to better performance, the 10-cycle PCR amplification of libraries was carried out using KAPA Hifi Polymerase. A post-PCR Agilent Bioanalyzer was used to quantify samples, followed by sample pooling and size-selection of pools using the LabChip XT Caliper. The multiplexed libraries were sequenced on the Illumina HiSeq 2000 for cohort 1 and HiSeq 4000 for cohorts 2–4 (75 bp paired-ends). Sequenced data underwent initial analysis and quality control (QC) on reads as standard. The sequencing depth was similar across samples, with 90% of samples passing final QC (see below) having 87.2–129.2 million reads.

### Proteomics

Proteomics analysis was performed on cartilage samples from 103 patients (Supplementary Data 7).

For cohort 1, all steps of protein digestion, 6-plex TMT labelling, peptide fractionation and LC-MS analysis on the Dionex Ultimate 3000 UHPLC system coupled with the high-resolution LTQ Orbitrap Velos mass spectrometer (Thermo Scientific), were previously described^12^ and are recapitulated in the Supplementary Methods. The sample preparation protocol formed the basis of processing for cohorts 2–4 using 10-plex TMT labelling and an Orbitrap Fusion Tribrid Mass Spectrometer (Thermo Scientific) with otherwise only minor alterations as described in the Supplementary Methods.

### Genotyping

We used Illumina HumanCoreExome-12v1-1 for genoting cohort 1 and Illumina InfiniumCoreExome-24v1-1 for genotyping cohort 2–4 patients.

### Quantification of RNA levels

We used samtools v1.3.1^37^ and biobambam v0.0.191^38^ to convert cram to fasq files after exclusion of reads that failed QC. We applied FastQC v0.11.5 to check sample quality^39^ and excluded nine samples (Supplementary Data 7).

We obtained transcript-level quantification using salmon 0.8.2^40^ (with–gcBias and –seqBias flags to account for potential biases) and the GRCh38 cDNA assembly release 87 downloaded from Ensembl [http://ftp.ensembl.org/pub/release-87/fasta/homo_sapiens/cdna/]. We used tximport^41^ to convert transcript-level to gene-level scaled transcripts per million (TPM) estimates, with estimates for 39,037 genes based on Ensembl gene IDs.

We excluded four samples due to low mapping rate (<80%), three samples due to non-European ancestry recorded in the clinic, 18 samples due to low RIN (<5), two samples as duplicates, eight samples due to abnormal gene read density plots (detected separately in cartilage and synovium for three cartilage and five synovium samples; all exclusions are listed in Supplementary Data 7).

The final gene expression dataset included 259 samples (Supplementary Fig. 1; 87 patients’ low-grade and 95 high-grade cartilage samples with 15,249 genes that showed counts per million (CPM) of ≥1 in ≥40 samples, and 77 patients’ synovium samples with 16,004 genes that showed CPM ≥1 in ≥20 samples).

### Quantification of protein levels

To carry out protein identification and quantification, we submitted the mass spectra to SequestHT search in Proteome Discoverer 2.1. The precursor mass tolerance was set at 30 ppm (Orbitrap Velos data, cohort 1) or 20 ppm (Fusion data, cohorts 2–4). For the CID spectra, we set the fragment ion mass tolerance to 0.5 Da; for the HCD spectra, to 0.02 Da. Spectra were searched for fully tryptic peptides with maximum two miss-cleavages and minimum length of six amino acids. We specified static modifications as TMT6plex at N-termimus, K and Carbamidomethyl at C; dynamic modifications included deamidation of N,Q and oxidation of M. For each peptide, we allowed for a maximum two different dynamic modifications with a maximum of two repetitions. We used the Percolator node to estimate peptide confidence. We set the peptide FDR at 1% and based validation on the *q* value and decoy database search. We searched all spectra against a UniProt fasta file that contained 20,165 reviewed human entries. The Reporter Ion Quantifier node included a TMT-6plex (Velos data, cohort 1) or TMT-10plex (Fusion data, cohorts 2–4) custom Quantification Method with integration window tolerance at 20 or 15 ppm, respectively. As integration methods, we used the Most Confident Centroid at the MS2 or MS3 level. We only used peptides uniquely belonging to protein groups for quantification.

We excluded samples from four patients due to non-European ancestry (Supplementary Data 7). The final dataset included low-grade and high-grade cartilage samples each from 99 patients, with 4801 proteins was observed in ≥30% of samples, and 1677 proteins in all samples, in line with the resolution depth of the isobaric labelling method employed. To account for protein loading, abundance values were normalised by the sum of all protein abundances in a given sample, then log2-transformed and quantile normalised.

### Genotype analysis and QC

Genotypes were called using GenCall (Illumina) and mapped to GRC37/hg19 using online tools (http://www.well.ox.ac.uk/~wrayner/strand/index.html). QC was carried out using the same method for both arrays. Briefly, we performed a pre-filtering step to exclude samples and variants with a call rate <90%. Sample QC included identity checks correlating the array genotypes to Fluidigm genotypes obtained at sample reception (no samples had a concordance <0.95). We excluded samples based on call rate <98%, heterozygosity distribution outliers performed using two different minor allele frequency (MAF) bins (≥1% MAF and <1% MAF) and sex discrepancies. We performed pairwise identity by descent (IBD) in PLINK^42,43^ after filtering out variants with MAF < 1% and carrying out linkage disequilibrium based pruning using R^2^ 0.2. We retained only patients with pairwise PI_HAT ≤ 0.2. To look at ethnicity we combined all patients from both arrays with data from the 1000 Genomes Project individuals (https://www.internationalgenome.org)^44^. We included overlapping variants only and conducting IBD, as described above, followed by multidimensional scaling using PLINK. Visual ethnic outliers were excluded following examination of the first two components. Variants were excluded if call rate <98% and/or Hardy–Weinberg *p* value (pHWE) <1 × 10^−4^. The final datasets contained 12 patients and 534,694 variants and 99 patients and 527,717 variants for cohorts 1 and 2–4, respectively.

Prior to imputation, all genotypes were combined into a single dataset containing 111 patients and 504,235 overlapping variants. Further QC was performed to exclude any variants with strand, position and allele frequency differences compared to the HRC panel^45^ using a HRC preparation checking tool (http://www.well.ox.ac.uk/~wrayner/tools/; v4.2.7). The resulting dataset contained 111 patients and 389,511 variants. We imputed up to HRC panel (v1.1 2016) using the Michigan imputation server (https://imputationserver.sph.umich.edu/index.html)^46^ with Eagle2 (v2.3) phasing. Post-HRC imputation we used a post-imputation data checking programme (http://www.well.ox.ac.uk/~wrayner/tools/Post-Imputation.html; v1.0.2) to visualise the results and we excluded variants with poor imputation quality (*R*^2^ < 0.3) and pHWE <1 × 10^−4^. We excluded two patients due to absence of RNA and protein data. The resulting final dataset contained 10,249,108 autosomal variants and 109 patients.

### Identification of *cis*-eQTLs

For each gene, we considered genetic variants within 1 Mb of the transcription start site (TSS) (definition see below), and followed a similar method to GTEx^9,47^ as follows.

For each tissue, we included only genes with ≥1 count per million in at least 20% samples and we normalised between samples using TMM (weighted trimmed mean of *M* values)^48^ implemented in edgeR^49^. To facilitate cartilage comparisons post-analysis, the previous two steps (exclusions of low expressed genes and the between sample normalisation) were performed with high-grade and low-grade cartilage samples combined. For each tissue separately, we then normalised across samples using an inverse normalisation transformation for each gene. To infer hidden factors associated with cohort, sequencing batch, or other technical differences, we applied probabilistic estimation of expression residuals (PEER)^50^ separately to each tissue (PEER C + + version with standard parameters from the R version, i.e. iteration = 1000, bound = 0.001, variance = 0.00001, Alpha a = 0.001, Alpha b = 0.1, Eps a = 0.1, Eps b = 10). We used the GTEx modified version of FastQTL^51^ (https://github.com/francois-a/fastqtl; v6p), which allows for minor allele count filtering, reporting of MAF and calculation of FDR. We determined the TSS for each gene using empirical transcript level expression information from synovium, high-grade and low-grade cartilage samples (see below) and defined the *cis*-mapping region to be 1 Mb in either direction from the TSS. We restricted the analysis to variants with minor allele count of at least 10 in a given tissue. Nominal *p* values for each gene-variant pair were based on linear regression, including 15 PEER factors for the given tissue, sex and genotype array as covariates. We then employed the adaptive permutation scheme with the—permute 1000 10000 option to generate empirical *p* values. Genes with significant eQTLs ('eGenes') were defined at the 5% Storey–Tibshirani FDR using the *q* values generated from the empirical *p* values^52^. For each eGene, significant eQTLs were defined as variants with nominal *p* value below the nominal *p* value threshold for that gene generated in FastQTL.

The normalised effect size (NES) of the eQTL is reported for the alternate allele according to GRC37/hg19.

### Identification of *cis*-pQTLs

We followed a similar protocol as for *cis*-eQTL analysis, also considering genetic variants within 1 Mb of the TSS (definition see below). For low-grade and high-grade cartilage, we included 1677 proteins that were measured across all samples. We normalised across samples using an inverse normalisation transformation for each gene separately in each tissue. To account for possible technical variation, we used PEER^50^ (with parameters as for the eQTL analysis above) and included 26 PEER factors, sex and genotype array as covariates using the GTEx modified version of FastQTL (https://github.com/francois-a/fastqtl; v6p). We used the TSS established for the eQTL analysis, yielding a unique mapping for 1461 proteins, which were then taken forward. For each protein, we considered variants within a 1 Mb region in either direction from the TSS, restricting further to minor allele count of 10 or higher. We then followed the same procedure as for *cis*-eQTLs to identify variant-protein pairs with significant *cis*-pQTL effects.

We carried out a protein–protein network analysis for the proteins with significant pQTL effects using the STRING v11.0 database^53^ (https://string-db.org/; see Supplementary Note 1).

### Sensitivity analysis for *cis*-eQTLs and *cis*-pQTLs

For both eQTLs and pQTLs, we verified that the results were robust by carrying out a sensitivity analysis including patient age and osteoarthritis joint (knee or hip) as covariates in addition to the PEER factors, sex and array (see Supplementary Note 1).

### Transcription start site (TSS) definition

To determine which transcript to use to define the TSS for each gene, we established the most abundant transcript for each gene. To have the same definition across all tissues, we analysed cartilage and synovium tissues jointly, considering 16,886 genes that passed quantification QC in at least one tissue (based on 15,249 genes in cartilage and 16,004 genes in synovium). For each transcript, we calculated the expression in each sample as scaled TPM using tximport^41^. For each gene, we then obtained the most abundant transcript in each tissue in each patient, and calculated the proportion of samples in which each transcript was the most abundant. The transcript that was the most abundant in the largest proportion of samples was used to define the TSS for the gene. For genes in which more than one transcript was the most abundant, we chose one of the most abundant transcripts at random to define the TSS.

Across all genes, 47.9% had the same most abundant transcript in at least 90% of samples in both cartilage and synovium; 71% of genes had the same most abundant transcript in 60% of samples in cartilage and synovium. We mapped the most abundant transcript (using the ENST identifier) to GRCh37 using ftp://ftp.ensembl.org/pub/grch37/release-87/gtf/homo_sapiens/Homo_sapiens.GRCh37.87.chr.gtf.gz. For 93 transcripts, ENSG identifiers differed between the builds, therefore genes were identified by a composite ID in the format GeneName(b37)_ENSG. We excluded 1940 transcripts with missing start or end positions (largely in patched genome build regions), and ~500 transcripts mapped to chromosomes X, Y or mitochondrial DNA, we established the TSS for 13,180 autosomal genes included in the cartilage and 13,708 genes included in the synovium molQTL analysis.

### Differential gene regulation in low-grade and high-grade cartilage

To identify differential regulation of gene expression between high- and low-grade cartilage, we used Meta-Tissue v0.5^54^ (downloaded from http://genetics.cs.ucla.edu/metatissue/index.html), which implements METASOFT^55^. The *m* value calculated by METASOFT for gene-variant pair in each tissue provides a posterior probability (*m* value) of an effect in that tissue. Consequently, we aimed to identify eQTLs present in one tissue (defined as *m* > 0.9), and absent in the other (defined as *m* < 0.1). We note that there were no *cis*-eQTLs present in both tissues (*m* > 0.9) with opposing direction of effect.

Meta-Tissue restricts covariates input to the same values for each patient across tissues, while different PEER covariates were provided for each tissue in the FastQTL analysis. Hence, for each tissue, we obtained residuals from regressing the normalised expression data on the 15 PEER factors, sex and array, then used the residuals as input for Meta-Tissue. We included genotype dosages based on both the low- and high-grade results for each analysis. We ran METASOFT using the default settings provided in the output script from Meta-Tissue. We only considered eQTLs that were identified in the FastQTL analysis in the appropriate tissue. To identify variants located in regulatory regions, we used Ensembl Variant Effect Predictor (http://grch37.ensembl.org/Homo_sapiens/Tools/VEP/). For 32 genes with differential QTLs, we considered gene annotations from several sources: using Gene Ontology biological process terms, through gene annotations in GOSeq^56^ (accessed on 31 May 2020), and from the summaries and functions sections on GeneCards gene pages (https://www.genecards.org, accessed 3 June 2020).

We also carried out a formal enrichment analysis of the 32 genes using GoSeq (see Supplementary Note 2).

### Co-localisation between molecular QTLs (molQTLs) and osteoarthritis GWAS associations

To examine co-localisation between molQTLs and GWAS associations, we used genome-wide summary statistics from the largest osteoarthritis meta-analysis to date, based on UK Biobank and arcOGEN data^8^. We analysed all 64 genome-wide significant signals using coloc^57^, separately for each tissue and omics level.

In the co-localisation analysis for each signal, we considered the region spanning 100 kb either side of the index variants. If that region overlapped any genes with significant *cis*-eQTLs or *cis*-pQTLs, we extended the region to encompass all variants included in the molQTL analysis for these genes. To formally obtain a posterior probability for co-localisation, we used coloc.fast (https://github.com/tobyjohnson/gtx/blob/526120435bb3e29c39fc71604eee03a371ec3753/R/coloc.R), a Bayesian statistical test which implements the coloc^57^ method. We used the default settings for coloc.fast. We considered a 80% posterior probability of GWAS and molQTL shared association at a single variant ('PP4 ≥ 0.8') to indicate evidence of co-localisation.

### Differential RNA expression between high-grade and low-grade cartilage

We tested differential expression of 15,249 genes between high-grade and low-grade cartilage using paired samples from 83 patients. To detect robust gene expression differences, we carried out analyses using different software packages as recommended in a landmark survey of best practices^58^, applying limma^59^, edgeR^60^ and DESeq2^61^. We also tested five analysis designs with different options to account for technical variation, including SVAseq^62^. In particular, we tested for differential expression using

(1) a paired analysis of intact and degraded samples (i.e. specifying patient ID as covariate);

(2) a paired analysis of intact and degraded samples, with ten additional covariates accounting for technical variation identified by SVAseq^62^;

(3) a paired analysis of intact and degraded samples, with ten RNA sequencing batches as covariates;

(4) an unpaired analysis of intact and degraded samples;

(5) an unpaired analysis of intact and degraded samples, with 19 additional covariates accounting for technical variation identified by SVAseq.

We tested for differential expression using the following R packages:

(i) limma^59^ (with lmFit and eBayes), after applying limma-voom^63^ to remove heteroscedasticity;

(ii) DESeq2^61^, separately with and without outlier filtering/replacement (minReplicatesForReplace = Inf, cooksCutoff = FALSE options);

(iii) edgeR^60^, using the likelihood ratio test (glmFit and glmLRT functions), and separately, using the F test (glmQLFit and glmQLFTest functions).

Here and elsewhere, we used Ensembl38p10 to identify genes with uniquely corresponding Ensembl gene ID and gene name (13,737 of 15,249 genes in the RNA data).

In each analysis design and method, we used a 5% FDR threshold to correct for multiple testing. As the final step, we applied a conservative approach and considered a gene ‘significantly differentially expressed' between low-grade and high-grade cartilage if it showed significant differential expression across all analysis designs and testing methods (2557 genes, including 2418 with uniquely corresponding Ensembl gene ID and gene name). As discussed in the Supplementary Note 3, for each analysis design, the number of significant genes at 5% FDR was similar across all tests.

Furthermore, we have performed a sensitivity analysis testing differential expression based on the patients in cohort 4 only and verified that results were highly consistent with the analysis of all patients (see Supplementary Note 3).

We used the pheatmap v1.0.12 function in R to plot the gene expression for the 20 genes with the highest absolute log-fold differences between high-grade and low-grade cartilage (selected from 290 genes with significant and concordant cross-omics differences, see Supplementary Methods).

### Differential protein abundance between high-grade and low-grade cartilage

We performed differential analysis for 4801 proteins that were measured in ≥30% of patients, applying limma^59^ to paired samples from 99 patients. Significance was defined at 5% FDR to correct for multiple testing, yielding 2233 proteins with significant differential abundance (2019 proteins with uniquely corresponding Ensembl gene ID and gene name).

As batch effects in proteomics data can be pervasive^64,65^, paired samples from any patient were always assayed in the same 6-plex (cohort 1) or 10-plex (cohorts 2–4). As a sensitivity analysis, we carried out a differential abundance analysis for the proteomics data with explicit adjustment for the plexes, to confirm that adjustment for patient effects was captured between-plex batch effects. For each protein, we calculated the log2 of normalised abundance values plus 1. We then obtained residuals from linear regression of these values on the 13 batches used in the proteomics data. These residuals were quantile normalised. We then used limma to test for differential abundance between low-grade and high-grade cartilage using the quantile normalised residuals, applying a paired design and the same method as in the main analysis. The results were highly similar to the main analysis (see Supplementary Note 4).

We used the pheatmap v1.0.12 function in R to plot the abundance of the 20 proteins with the highest absolute log-fold differences between high-grade and low-grade cartilage (selected from 290 genes with significant and concordant cross-omics differences, see Supplementary Methods).

### Replication of differences between high-grade and low-grade cartilage

We used an independent dataset from the RAAK study (35 osteoarthritis patients, of which 28 knee, 7 hip osteoarthritis) to replicate the molecular differences between high-grade and low-grade cartilage. The RAAK dataset and analysis were taken from a previous publication^15^. Briefly, mRNA samples were sequenced on Illumina HiSeq 2000/4000 (paired-end 2 × 100 bp RNA-sequencing), aligned using GSNAP^66^, and quantified using HTSeq^67^. We used the differential expression analysis as originally published^15^, based on DESeq2, with removal of batch effects using the function removeBatchEffect from the limma R package, then application of a general linear model assuming a negative binomial distribution and a paired Wald-test between preserved and lesioned OA cartilage samples. The data were downloaded from the Github repository https://git.lumc.nl/rcoutinhodealmeida/miRNAmRNA on 20 June 2020. The analysis as published included 20,165 genes, of which 2387 were differentially expressed at 5% FDR. Of these genes, 12,663 had uniquely mapping Ensembl gene ID and gene name and were also assayed in our data (including 1830 genes with significant differential expression at 5% FDR in the RAAK study). For the genes with RNA-level, protein-level or cross-omics level differential expression in the discovery analysis above, we calculated the proportion with concordant direction of effect in the RAAK data, and applied Fisher’s test to determine whether this proportion was higher than for other genes (RNA-level: compared to all genes not differentially expressed; protein- and cross-omics level: compared to all genes not differentially expressed, but assayed in proteomics). We also calculated the proportion of genes with *p* < 0.05 and FDR < 5% in the RAAK analysis, and the proportion of these with concordant direction of effect.

### Pathway associations for differences between high-grade and low-grade cartilage

To identify the biological processes with significant molecular differences between high-grade and low-grade cartilage, we carried out gene set enrichment analyses based on the differential expression on RNA, protein and cross-omics levels. We tested for association of the differentially expressed (DE) genes on RNA and/or protein levels at 5% FDR, with robustness checks using more stringent FDR thresholds (1, 0.5 and 0.1%). We restricted this analysis to genes with unique mapping between Ensembl gene ID and Gene Name in Ensembl38p10, and Ensembl gene ID and Entrez ID in HUGO (https://www.genenames.org/ accessed 05/03/2018; 13,094 genes measured on RNA level, 4390 genes measured on protein level, 4387 genes measured on both RNA and protein levels).

We applied Signalling Pathway Impact Analysis (SPIA)^68^ to test for association with KEGG signalling pathways. SPIA combines enrichment *p* values with perturbation impact on the pathway based on log-fold differences of the DE genes; perturbation *p* values are obtained by bootstrapping. Enrichment and perturbation *p* values were combined using a normal inversion method which only gives low *p* values when both over-representation and pathway impact *p* values are low (function option combine = 'norminv'). Significance of pathway association was defined as a threshold of 5% FDR applied to the combined *p* values in each analysis. For the analysis of genes DE on both RNA and protein levels, we carried out tests using the log-fold differences from the RNA data (based on the limma analysis with paired samples and SVAseq covariates), and separately, from the protein data.

We also tested enrichment in Gene Ontology terms using GOseq^56^, separately for genes with higher or lower expression in high-grade compared to low-grade cartilage. We accounted for gene length (pwf function options ‘hg19' and ‘geneSymbol'). Significance was defined as a threshold of 5% FDR in each analysis. The results showed broad agreement with the results of the SPIA analysis (see Supplementary Note 5 and Supplementary Data 3).

### Identification of genes with osteoarthritis GWAS gene-level association

From the recent UK Biobank and arcOGEN GWAS meta-analysis^8^, we obtained the results of a gene-level analysis for each of the four osteoarthritis phenotypes (self-reported plus hospital diagnosed, hospital diagnosed knee or hip, hospital diagnosed knee, hospital diagnosed hip), as described in the GWAS paper. Briefly, this analysis used MAGMA v1.06^69^ and was based on the mean SNP log-*p*-value in the gene, accounting for LD.

To calculate the effective number of tests across phenotypes, we calculated the correlation matrix between the gene *p* values for the four osteoarthritis phenotypes, and obtained the eigenvalues of this matrix. The effective number of tests *N*_eff_ for phenotypes was then calculated as $N_{eff} = N - \mathop {\sum}\nolimits_\lambda {I(\lambda > 1) \ast (\lambda - 1)}$, where *N* = 4 is the number of phenotypes, and *λ* denotes the eigenvalues. Across the Pearson and Spearman correlation matrices, we obtained *N*_eff_ < 2.65. With 18,449 genes per phenotype, the significance threshold for gene-level *p* values across genes and phenotypes was thus set as 0.05/(18,449 × 2.65) = 1.02 × 10^−6^.

After accounting for the effective number of tests across phenotypes and genes using a Bonferroni correction, 320 of 18,449 genes showed significant association with at least one phenotype. Of these genes, 238 genes were compared between low-grade and high-grade cartilage on at least one omics level and had uniquely corresponding Ensembl gene ID and gene name.

### ConnectivityMap analysis

To identify opportunities for drug repurposing, we used ConnectivityMap^17^ to identify compounds and perturbagen classes (PCLs) that could possibly reverse the differences identified between high-grade and low-grade cartilage. Using the online interface clue.io (accessed 3 March 2019), we submitted the 148 genes with significantly higher expression on both RNA and protein level to calculate a ‘tau’ connectivity score to gene expression signatures experimentally induced by various perturbations in nine cell lines. A positive tau score indicates similarity between the gene expression signature of a perturbation and the submitted query (i.e. upregulation of the genes with higher expression in high-grade compared to low-grade cartilage). A negative tau score indicates that gene expression signature of a perturbation opposes the submitted query (i.e. downregulation of the genes with higher expression in high-grade compared to low-grade cartilage). Recommended thresholds for further consideration of results are tau of at least 90, or below −90, respectively (https://clue.io/connectopedia/connectivity_scores, accessed 3 March 2019). A total of 2837 compound and 171 PCL perturbations were evaluated in clue.io. We shortlisted perturbations where both the summary tau and the median tau across cell lines were higher than 90 or lower than −90 for PCLs, with more conservative thresholds of higher than 95 or lower than −95 for compounds. The clue.io platform also contained perturbation data from 3799 gene knock-down and 2160 over-expression experiments (with 2111 genes in both, i.e. 3848 genes total). These data were used to shortlist genes where both the summary and median tau were higher than 95 or lower than −95.

### Reporting Summary

Further information on research design is available in the Nature Research Reporting Summary linked to this article.

## Supplementary information

Supplementary Information

Peer Review File

Reporting Summary

Description of Additional Supplementary Files

Supplementary Data 1

Supplementary Data 2

Supplementary Data 3

Supplementary Data 4

Supplementary Data 5

Supplementary Data 6

Supplementary Data 7

## Data Availability

The RNA sequencing data reported in this paper have been deposited to the EGA (accession numbers EGAD00001005215, EGAD00001003355, EGAD00001003354, EGAD00001001331. The proteomics data reported in this paper have been deposited to PRIDE (accession numbers PXD014666, PXD006673, PXD002014. The genotype data reported in this paper have been deposited to the EGA (accession numbers EGAD00010001746, EGAD00010001285, EGAD00010001292, EGAD00010000722. Data from the 1000 Genomes Project are publicly available (https://www.internationalgenome.org). We also used publicly available data on osteoarthritis differential gene expression from the RAAK Study (https://git.lumc.nl/rcoutinhodealmeida/miRNAmRNA, accessed 20 June 2020). Further data including the TSS information, all significant molQTLs and full co-localisation results, can be obtained online from https://hmgubox.helmholtz-muenchen.de/d/fc1fcf65a6724152b7f9/. The full molecular QTL data and molecular differences between high-grade and low-grade cartilage are available through the Downloads page of the Musculoskelatal Knowledge Portal (mskkp.org).

## Code availability

All software used in this study is available from free repositories or from manufacturers as referenced in the Methods section.
