# Supplementary material for: A molecular quantitative trait locus map for osteoarthritis
Source: Nat Commun. 2021 Feb 26;12:1309. doi: 10.1038/s41467-021-21593-7 (PMC7910531; doi:10.1038/s41467-021-21593-7)
Supplement: Supplementary file 1 — Supplementary Information [file 41467_2021_21593_MOESM1_ESM.pdf]

A molecular quantitative trait locus map for osteoarthritis

Julia Steinberg[1,2,3,4], Lorraine Southam[1,3], Theodoros I Roumeliotis[3,5], Matthew J Clark[6], Raveen L Jayasuriya[6], Diane Swift[6], Karan M Shah[6], Natalie C Butterfield[7], Roger A Brooks[8], Andrew W McCaskie[8], JH Duncan Bassett[7], Graham R Williams[7], Jyoti S Choudhary[3,5], J Mark Wilkinson[6,9,11], Eleftheria Zeggini[1,3,10,11]

[1] Institute of Translational Genomics, Helmholtz Zentrum München – German Research Center for Environmental Health, 85764 Neuherberg, Germany

[2] Cancer Research Division, Cancer Council NSW, Sydney, New South Wales 2011, Australia

[3] Wellcome Sanger Institute, Hinxton CB10 1SA, United Kingdom

[4] School of Public Health, The University of Sydney, Sydney, New South Wales 2006, Australia

[5] The Institute of Cancer Research, London SW7 3RP, UK

[6] Department of Oncology and Metabolism, University of Sheffield, Sheffield S10 2RX, UK

[7] Molecular Endocrinology Laboratory, Department of Metabolism, Digestion and Reproduction, Imperial College London, London W12 ONN, UK

[8] Division of Trauma & Orthopaedic Surgery, Department of Surgery, University of Cambridge, Cambridge CB2 2QQ, UK

[9] Centre for Integrated Research into Musculoskeletal Ageing and Sheffield Healthy Lifespan Institute, University of Sheffield, Sheffield S10 2TN, UK

[10] TUM School of Medicine, Technical University of Munich and Klinikum Rechts der Isar, Munich, Germany

[11] These authors contributed equally

Correspondence to: j.m.wilkinson@sheffield.ac.uk; eleftheria.zeggini@helmholtz-muenchen.de

Supplementary Information

Table of Contents

Supplementary Notes **3**
Supplementary Note 1: Identification of *cis*-eQTLs and *cis*-pQTLs in osteoarthritis tissues 3
Supplementary Note 2: Differential eQTLs between low-grade and high-grade cartilage 4
Supplementary Note 3: Differential gene expression between high-grade and low-grade cartilage 5
Supplementary Note 4: Differential protein abundance between high-grade and low-grade cartilage .. 7
Supplementary Note 5: Pathway associations for differences between high-grade and low-grade cartilage 8
Supplementary Note 6: Candidate therapeutic compounds 9
Supplementary Methods **10**
DNA, RNA and protein extraction: cohort 3 10
Proteomics: cohort 1 10
Proteomics: cohorts 2-4 13
Supplementary Figures **16**
Data available online **29**
Supplementary References **30**

Supplementary Notes

Supplementary Note 1: Identification of *cis*-eQTLs and *cis*-pQTLs in osteoarthritis tissues

To verify that the molQTL results were robust, we carried out a sensitivity analysis including patient age and osteoarthritis joint (knee or hip) as covariates in addition to the PEER factors, sex and array. In each tissue, ≥89.8% of eGenes and >82% of pGenes significant in the main analysis also remained significant at 5% FDR in the sensitivity analysis, with a very strong correlation of normalized effect sizes reported for eGene and pGene variants identified across both analyses (Pearson $r \geq 0.988$, $P<10^{-15}$).

We note that we detected a smaller proportion of genes with pQTLs (38 of 1,677 proteins in cartilage, 2.6%) than with eQTLs (1,569 of 15,249 in cartilage, 10.3%). Generally, protein expression is influenced by RNA levels as well as translational and post-translational mechanisms, so it is possible that the regulatory effects from genetic variants are diminished. A recent study of brain samples also found fewer genes with pQTLs than with eQTLs (14.7% versus 10.7% of 5,743 genes profiled on both assays)[1]. Moreover, differences in the detected numbers of eQTLs and pQTLs are also likely to be influenced by technological differences between the RNA sequencing and proteomics platforms.

We investigated whether variants that are significant pQTLs also exhibit evidence of eQTL effects (Supplementary Fig. 3). In low-grade cartilage, we detected 2871 pQTL variants, of which 2866 were also present in the low-grade cartilage eQTL analysis. Of these variants,

2577 (90%) have the same direction of effect on gene expression and protein levels, and 1295 (50%) were also significant eQTLs in low-grade cartilage at 5% FDR.

In high-grade cartilage, we detected 1971 pQTL variants, of which 1969 variants were also present in the high-grade eQTL analysis. Of these 1786 (91%) have the same direction of effect on gene expression and protein levels, and 1133 (63%) were also significant eQTLs in high-grade cartilage at 5% FDR.

We carried out a protein-protein network analysis to identify known and potential connections between the 38 proteins with significant pQTLs identified in cartilage, using the STRING v11 database[2] (https://string-db.org/). This identified 21 connections between the proteins (Supplementary Fig. 4). While this is significantly higher than the number of connections between 38 randomly selected proteins (STRING $p=3.08 \times 10^{-8}$), the formal network analysis does not account for the abundance level of proteins, which impacts the power to detect both pQTLs and functional interactions.

Supplementary Note 2: Differential eQTLs between low-grade and high-grade cartilage

Among 32 genes with differential eQTLs, 20 genes showed strong evidence for specific regulatory effects present in high-grade, but not low-grade cartilage; the remaining 12 genes showed strong evidence for specific regulatory effects present in low-grade, but not high-grade cartilage. We tested whether the two groups differed in genes annotated to “regulation of gene expression”, “nervous system development”, “response to stress”, “immune response”, “cell adhesion” and “catabolic processes” (based on annotations from GoSeq and GeneCards, see Methods). We did not find any significant associations (Fisher’s

test p>0.06), which could be due to a broader rewiring of regulatory processes (with some losses and some gains of genetic regulatory effects), and/or due to the small numbers of genes in the enrichment analysis.

We also tested whether genes with differential eQTL were enriched in any GeneOntology annotations with 20-200 genes using GoSeq, compared to all 1569 gene with an eQTL in any cartilage tissue. We separately analysed all 32 genes, as well as 20 respectively 12 genes based on tissue with observed differential eQTL effect, restricting the analysis to genes with unique Ensembl gene ID, gene symbol and Entrez gene ID. Again, we did not detect any significant associations (FDR>5%).

We note that this approach is simplified due to not accounting for several factors that influence the power to detect eQTLs, including the expression levels of genes, the number of SNPs within the gene region, their LD pattern and minor allele frequency. Given the limited power to detect associations based on a small number of genes, we have left more in-depth enrichment analyses of genes with differential eQTLs for future work based on larger sample sizes.

Supplementary Note 3: Differential gene expression between high-grade and low-grade cartilage

We compared the differential expression results between the 5 analysis designs as well as 5 different tests (limma, DESeq2 with and without outlier filtering, edgeR likelihood ratio test and edgeR F test). For each analysis design, the number of significant genes at 5% FDR was similar across all tests:

1) 7119-7487 genes for the paired analysis of intact and degraded samples (i.e. specifying patient ID as covariate);
2) 5774-6126 genes for the paired analysis of intact and degraded samples, with 10 additional covariates accounting for technical variation identified by SVAseq;
3) 4005-4286 genes for the paired analysis of intact and degraded samples, with 10 RNA sequencing batches as covariates;
4) 3305-3364 genes for the unpaired analysis of intact and degraded samples;
5) 6852-7027 genes for the unpaired analysis of intact and degraded samples, with 19 additional covariates accounting for technical variation identified by SVAseq.

The results agree with the higher power expected in a paired analysis compared to an unpaired analysis. Conservatively, we considered a gene "significantly differentially expressed" between low-grade and high-grade cartilage if it showed significant differential expression across all analysis designs and testing methods (2,557 genes).

We also carried out a sensitivity analysis of differential expression using the subset of patients from cohort 4 only (47 patients with paired high-grade and low-grade cartilage samples). We included the same covariates as in the main analysis and applied the same analysis designs and tests for differential expression. Of the 2,557 genes with significant differences in the main analysis, 1,979 (77.4%) were also significant across all analysis designs and approaches in the sensitivity analysis. There was a high correlation of effect estimates between the main and sensitivity analysis. For example, in the paired analysis of intact and degraded samples, with 10 additional covariates accounting for technical variation identified by SVAseq, using limma, the Pearson correlation of log-fold-differences

was r=0.82 across all 15,249 genes, and even higher at r=0.96 across the 2,557 genes with significant differences in the main analysis.

Supplementary Note 4: Differential protein abundance between high-grade and low-grade cartilage

As batch effects in proteomics data can be pervasive[3,4], paired samples from any patient were always assayed in the same 6-plex (cohort 1) or 10-plex (cohorts 2-4). As a sensitivity analysis, we carried out a differential abundance analysis for the proteomics data with explicit adjustment for the plexes (see Methods). The results were highly similar to the main analysis. In particular, the correlation of protein log-fold-changes between the sensitivity and main analyses was very high (Pearson r=0.98 and Spearman ρ=0.97, both $P<10^{-10}$). Of the 2,233 proteins with FDR<5% in the main analysis, all had a concordant direction of effect in the sensitivity analyses, and 1,796 (80.4%) were also significant at 5% FDR in the sensitivity analysis. As the low-grade and high-grade samples from the same patient were assayed in the same 6-plex or 10-plex, the adjustment for patient effects in the main analysis is expected to capture batch effects, as indeed confirmed by this sensitivity analysis.

Generally, many past studies have found discrepancies between mRNA and protein levels due to different post-transcriptional and translational regulation and different rates of degradation (e.g. [5]). This gives rise to different relative expression and abundance levels as assessed by RNA-seq and quantitative proteomics, and different technical noise for the two omics levels. Thus, the power to detect differences on RNA and protein level would also be different, and affect which differences are statistically significant at the 5% FDR level.

Of the 4390 genes measured on both RNA and protein level, 409 genes were differentially expressed on both omics levels, 397 genes on RNA-level only, 1610 genes on protein-level only, and 1974 not on either omics level. That overlap in detected cross-omics molecular differences is significant (Fisher's p-value 0.0029).

We also found strong evidence for concordant direction of expression changes across the two omics levels, with a correlation of r=0.63 (P<$1.0\text{x}10^{-17}$) between the RNA- and protein-level effect sizes for genes with cross-omics changes (Supplementary Fig. 8b), providing internal cross-validation for the approaches used.

Supplementary Note 5: Pathway associations for differences between high-grade and low-grade cartilage

In particular, significant associations in the Signalling Pathway Impact Analysis (SPIA) include the activation of the "ECM-receptor interaction" in high-grade compared to low-grade cartilage (identified across RNA-level, protein-level, and cross-omics DE genes), with activations of the "Focal adhesion", "Lysosome", and "Cytokine-cytokine receptor interaction" pathways identified based on the RNA data (Supplementary Fig. 10, Supplementary Data 3).

These associations agree with enrichment analyses based on Gene Ontology (GO) terms in GOseq, carried out separately for genes with higher or lower expression in high-grade cartilage compared to low-grade cartilage. Here, we identified associations with terms related to "extracellular space" for genes with higher expression on RNA-level, and separately, protein-level, and across omics levels. Genes with higher expression on RNA-level also showed significant associations with terms related to "plasma membrane", "vesicle", "cell adhesion", "cell communication", and "signaling" (Supplementary Data 3).

Protein-level differentially expressed genes showed enrichments for “extracellular region” and related terms among genes with both higher and lower expression; genes with lower expression also showed associations with “small molecule metabolic process”, “vesicle”, “ECM structural constituent“, and “cofactor binding” (Supplementary Data 3).

Supplementary Note 6: Candidate therapeutic compounds

We note that one of the shortlisted oestrogen receptor agonists, alpha-estradiol, targets *KCNMA1*, coding for the pore-forming alpha subunit of a calcium-sensitive potassium channel that demonstrated significantly lower gene expression and protein abundance in high-grade cartilage.

A conditional knock-down of *Vegf* attenuates surgically-induced osteoarthritis in mice, with intra-articular anti-VEGF antibodies as well as oral administration of the VEGFR2 kinase inhibitor Vandetanib suppressing osteoarthritis progression[6].

RHO-kinase-inhibitor-III[rockout] is a rho associated kinase inhibitor. In a rat model of osteoarthritis, a rho kinase inhibitor was found to reduce knee cartilage damage[7].

Supplementary Methods

DNA, RNA and protein extraction: cohort 3

For samples from cohort 3, cells were lysed in 350μl RLT buffer containing 3.5μl β-mercaptoethanol and centrifuged in a Qiashredder column (Qiagen) for 2 min at 20,800g. RNA, DNA and protein were purified using a series of column based centrifugation steps and RNA was eluted in 34 μl RNase-free water, DNA in 100 μl EB buffer and the protein pellet solubilised in 100μl 10% sodium dodecyl sulphate in PBS. DNA and RNA preparations were re-precipitated to remove buffer salts and to concentrate the final product: 20μl 3M sodium acetate, pH 5.5 (Ambion) was added per 100μl of DNA or RNA solution and mixed gently. Then, 283μl of molecular grade ethanol (Sigma) was added per 100μl of DNA or RNA solution to precipitate the nucleic acids and the mixture placed at -20°C overnight. Samples were centrigfuged at 20,800g for 30 minutes and the supernatant removed, the pellet was washed with 900μl 70% ethanol and the nucleic acids re-pelleted by spinning at 20,800g for 10 minutes. Nucleic acids were resuspended in 32μl DNase/RNase free water.

Proteomics: cohort 1

Protein Digestion and TMT Labeling

The protein content of each sample was precipitated by the addition of 30 μL TCA 8 M at 4 °C for 30 min. The protein pellets were washed twice with ice cold acetone and finally re-suspended in 40 μL 0.1 M triethylammonium bicarbonate, 0.05% SDS with pulsedprobe sonication. Protein concentration was measured with Quick Start Bradford Protein Assay (Bio-Rad) according to manufacturer's instructions. Aliquots containing 30 μg of total protein were prepared for trypsin digestion. Cysteine disulfide bonds were reduced by the

addition of 2 µL 50 mM tris-2-carboxymethyl phosphine (TCEP) followed by 1 h incubation in heating block at 60 °C. Cysteine residues were blocked by the addition of 1 µL 200 mM freshly prepared Iodoacetamide (IAA) solution and 30 min incubation at room temperature in dark. Trypsin (Pierce, MS grade) solution was added at a final concentration 70 ng/µL to each sample for overnight digestion. After proteolysis the peptide samples were diluted up to 100 µL with 0.1 M TEAB buffer. A 41 µL volume of anhydrous acetonitrile was added to each TMT 6-plex reagent (Thermo Scientific) vial and after vortex mixing the content of each TMT vial was transferred to each sample tube. Labeling reaction was quenched with 8 µL 5% hydroxylamine for 15 min after 1 h incubation at room temperature. Samples were pooled and the mixture was dried with speedvac concentrator and stored at -20 °C until the high-pH Reverse Phase (RP) fractionation.

Peptide fractionation

Offline peptide fractionation based on high pH Reverse Phase (RP) chromatography was performed using the Waters, XBridge C18 column (2.1 x 150 mm, 3.5 µm, 120 Å) on a Dionex Ultimate 3000 HPLC system equipped with autosampler. Mobile phase (A) was composed of 0.1% ammonium hydroxide and mobile phase (B) was composed of 100% acetonitrile, 0.1% ammonium hydroxide. The TMT labelled peptide mixture was reconstituted in 100 µL mobile phase (A), centrifuged and injected for fractionation. The multi-step gradient elution method at 0.2 mL/min was as follows: for 5 minutes isocratic at 5% (B), for 35 min gradient to 35% (B), gradient to 80% (B) in 5 min, isocratic for 5 minutes and re-equilibration to 5% (B). Signal was recorded at 280 nm and fractions were collected in a time dependent manner every one minute. The collected fractions were dried with SpeedVac concentrator and stored at -20 °C until the LC-MS analysis.

LC-MS Analysis

LC-MS analysis was performed on the Dionex Ultimate 3000 UHPLC system coupled with the high-resolution LTQ Orbitrap Velos mass spectrometer (Thermo Scientific). Each peptide fraction was reconstituted in 40 µL 0.1% formic acid and a volume of 10 µL was loaded to the Acclaim PepMap 100, 100 µm × 2 cm C18, 5 µm, 100 Å trapping column with a user modified injection method at 10 µL/min flow rate. The sample was then subjected to a multi-step gradient elutionon the Acclaim PepMap RSLC (75 µm × 50 cm, 2 µm, 100 Å) C18 capillary column (Dionex) retrofitted to an electrospray emitter (New Objective, FS360-20-10-N-20-C12) at 45 °C. Mobile phase (A) was composed of 96% H2O, 4% DMSO, 0.1% formic acid and mobile phase (B) was composed of 80% acetonitrile, 16% H2O, 4% DMSO, 0.1% formic acid. The gradient separation method at flow rate 300 nL/min was as follows: for 95 min gradient to 45% B, for 5 min up to 95% B, for 8 min isocratic at 95% B, re-equilibration to 5% B in 2 min, for 10 min isocratic at 5% B.

The ten most abundant multiply charged precursors within 380 -1500 *m/z* were selected with FT mass resolution of 30,000 and isolated for HCD fragmentation with isolation width 1.2 Th. Normalized collision energy was set at 40 and the activation time was 0.1 ms for one microscan. Tandem mass spectra were acquired with FT resolution of 7,500 and targeted precursors were dynamically excluded for further isolation and activation for 40 seconds with 10 ppm mass tolerance. FT max ion time for full MS experiments was set at 200 ms and FT MSn max ion time was set at 100 ms. The AGC target vales were 3×10e6 for full FTMS and 1×10e5 for MSn FTMS. The DMSO signal at m/z 401.922718 was used as a lock mass.

Proteomics: cohorts 2-4

Protein Digestion and TMT Labeling

The protein content of each sample was precipitated by the addition of 30 μL TCA 8 M at 4 °C for 30 min. The protein pellets were washed twice with ice cold acetone and finally re-suspended in 40 μL 0.1 M triethylammonium bicarbonate, 0.1% SDS with pulsed probe sonication. Equal aliquots containing at least 10 μg of total protein were reduced with 5 mM TCEP for 1h at 60 °C, alkylated with 10 mM Iodoacetamide and subjected to overnight trypsin (70 ng/μL) digestion. TMT 10-plex (Thermo Scientific) labelling was performed according to manufacturer's instructions at equal amounts of tryptic digests. Samples were pooled and the mixture was dried with speedvac concentrator and stored at -20 °C until the peptide fractionation.

Peptide fractionation

Offline peptide fractionation was based on high pH Reverse Phase (RP) chromatography using the Waters, XBridge C18 column (2.1 x 150 mm, 3.5 μm) on a Dionex Ultimate 3000 HPLC system. Mobile phase A was 0.1% ammonium hydroxide and mobile phase B 100% acetonitrile, 0.1% ammonium hydroxide. The TMT labelled peptide mixture was dissolved in 100 μL mobile phase A, centrifuged and injected for fractionation. The gradient elution method at 0.2 mL/min included the following steps: 5 minutes isocratic at 5% B, for 35 min gradient to 35% B, gradient to 80% B in 5 min, isocratic for 5 minutes and re-equilibration to 5% B. Signal was recorded at 280 nm and fractions were collected every one minute. For cohort 4, peptide fractionation was performed on reversed-phase OASIS HLB cartridges at high pH and up to 9 fractions (10-25% acetonitrile elution steps) were collected for each set.

The collected fractions were dried with SpeedVac concentrator and stored at -20 °C until the LC-MS analysis.

LC-MS Analysis

LC-MS analysis was performed on the Dionex Ultimate 3000 UHPLC system coupled with the Orbitrap Fusion Tribrid Mass Spectrometer (Thermo Scientific). Each peptide fraction was reconstituted in 40 μL 0.1% formic acid and a volume of 7 μL was loaded to the Acclaim PepMap 100, 100 μm × 2 cm C18, 5 μm, 100 Ȧ trapping column with the μlPickUp mode at 10 μL/min flow rate. The sample was then analysed with a gradient elution on the Acclaim PepMap RSLC (75 μm × 50 cm, 2 μm, 100 Å) C18 capillary column retrofitted to an electrospray emitter (New Objective, FS360-20-10-D-20) at 45 °C. Mobile phase A was 0.1% formic acid and mobile phase B was 80% acetonitrile, 0.1% formic acid. The gradient method at flow rate 300 nL/min was: for 90 min gradient to 38% B, for 5 min up to 95% B, for 13 min isocratic at 95% B, re-equilibration to 5% B in 2 min, for 10 min isocratic at 10% B. Precursors were selected with 120k mass resolution, AGC 3×10^5 and IT 100 ms in the top speed mode within 3 sec and were targeted for CID fragmentation with quadrupole isolation width 1.2 Th. Collision energy was set at 35% with AGC 1×10^4 and IT 35 ms. MS3 quantification spectra were acquired with further HCD fragmentation of the top 10 most abundant CID fragments isolated with Synchronous Precursor Selection (SPS) excluding neutral losses of maximum m/z 18. Iontrap isolation width was set at 0.7 Th for MS1 isolation, collision energy was applied at 55% and the AGC setting was at 6×10^4 with 100 ms IT. The HCD MS3 spectra were acquired within 110-400 m/z with 60k resolution. Targeted precursors were dynamically excluded for further isolation and activation for 45 seconds with 7 ppm mass tolerance. Cohort 4 were analyzed at the MS2 level with a top15 HCD

method (CE 40%, 50k resolution) and a maximum precursor intensity threshold of 5×10^7 using the same MS1 parameters as above in a 360 min gradient.

Heatmaps of molecular differences between high-grade and low-grade cartilage

We used the pheatmap v1.0.12 function in R to plot heatmaps of gene expression and protein abundance values. Among the 290 genes with significant and concordant cross-omics differences, we selected the top 20 genes and top 20 proteins with the highest absolute log-fold-differences between high-grade and low-grade cartilage. For gene expression, this was based on the paired-sample analysis with limma including the 10 technical covariates inferred by SVseq. Analogously to the differential gene expression and protein abundance analysis, we then regressed out patient-level effects and 10 technical covariates for gene expression, and the patient-level effects for the protein abundance (as paired samples were always assayed in the same batch).

Supplementary Figures

Omics level	Protein			RNA				Total
Tissue	High-grade cartilage	Low-grade cartilage	High- and low-grade cartilage	High-grade cartilage	Low-grade cartilage	High- and low-grade cartilage	Synovium	
Number of patients	99	99	99	95	87	83	77	109

Supplementary Figure 1. Large-scale multi-omics characterisation of osteoarthritis disease tissue: number of patients with data for each tissue and omics type after quality control. All 109 patients also have genome-wide genotype data.

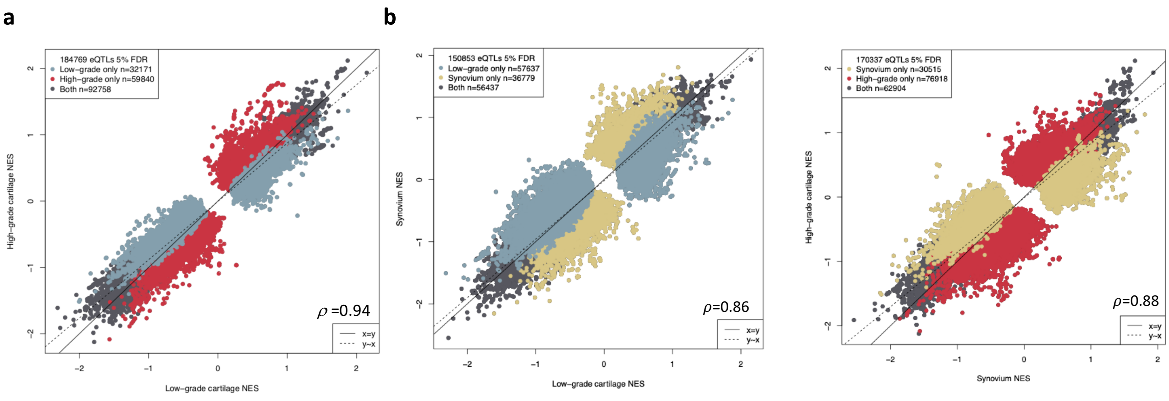

Supplementary Figure 2. *cis*-eQTLs in osteoarthritis disease tissue.

a, High correlation of eQTL normalized effect sizes (NES) between low-grade and high-grade cartilage. Inset: Spearman correlation between NES effect sizes across all eQTLs. The direction of effect was concordant across all *cis*-eQTLs detected in both low- and high-grade cartilage (92,758 variant-gene pairs).

b, High correlation of eQTL normalized effect sizes (NES) between low-grade cartilage and synovium (left), and between high-grade cartilage and synovium (right). Inset: Spearman correlation between NES effect sizes across all eQTLs.

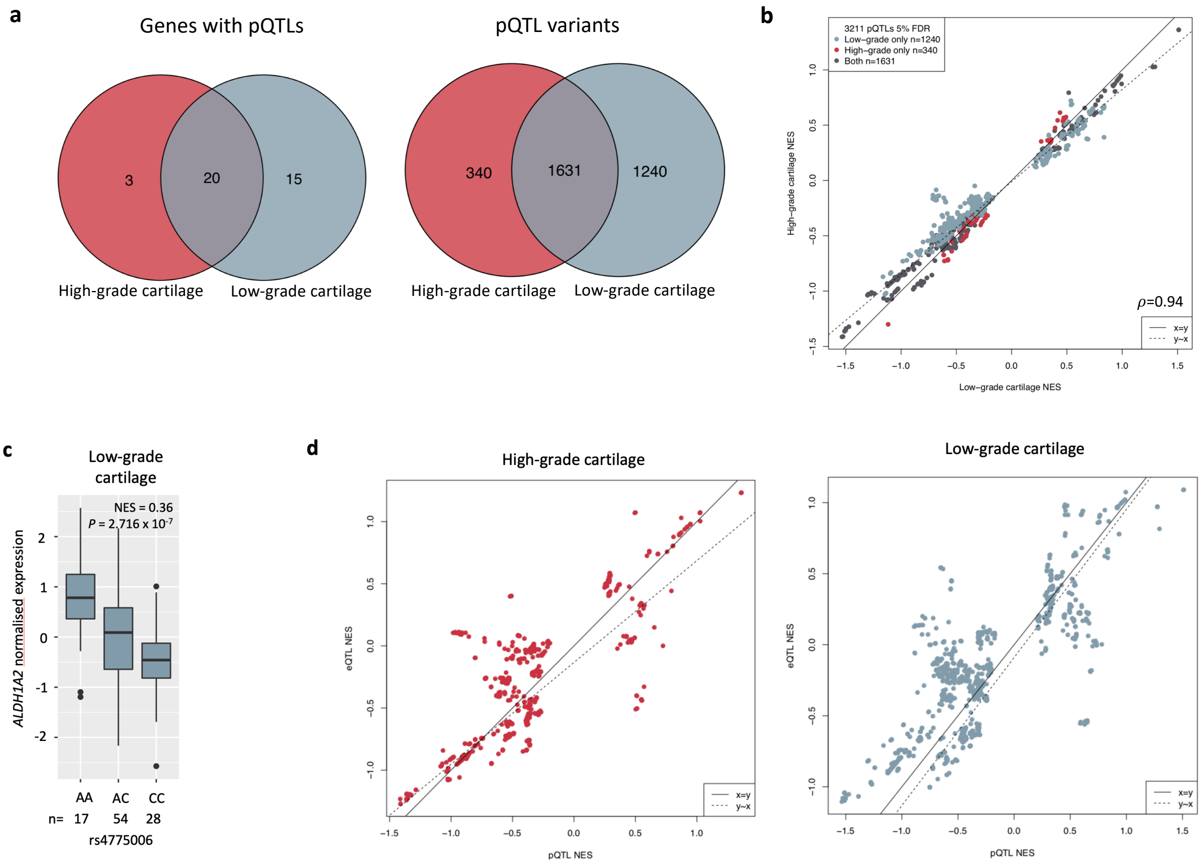

Supplementary Figure 3. *cis*-pQTLs in osteoarthritis disease tissue.

- **a,** pQTL overlap between tissues, for a total of 38 genes with a least one pQTL (left) and 3,211 pQTL protein-variant pairs (right).
- **b,** High correlation of pQTL NES between low-grade and high-grade cartilage. Inset: Spearman correlation between NES effect sizes across all eQTLs.
- **c,** An example of a pQTL effect for *ALDH1A2*, for which we detected colocalisation of GWAS association and pQTL effect in low-grade cartilage. Genotype-dependent protein abundance for the GWAS index variant is displayed. The boxplots show expression (residuals after regressing the normalised expression data on the 15 PEER factors, sex and array) at 25th to 75th percentiles, with centre at the median and whiskers extend to 1.5 times the interquartile range. NES: FastQTL normalised effect

size; *P*: FastQTL association *P*-value, n: number of individuals included in the analysis for each genotype.

d, Correlation of pQTL NES and eQTL NES in high-grade and low-grade cartilage

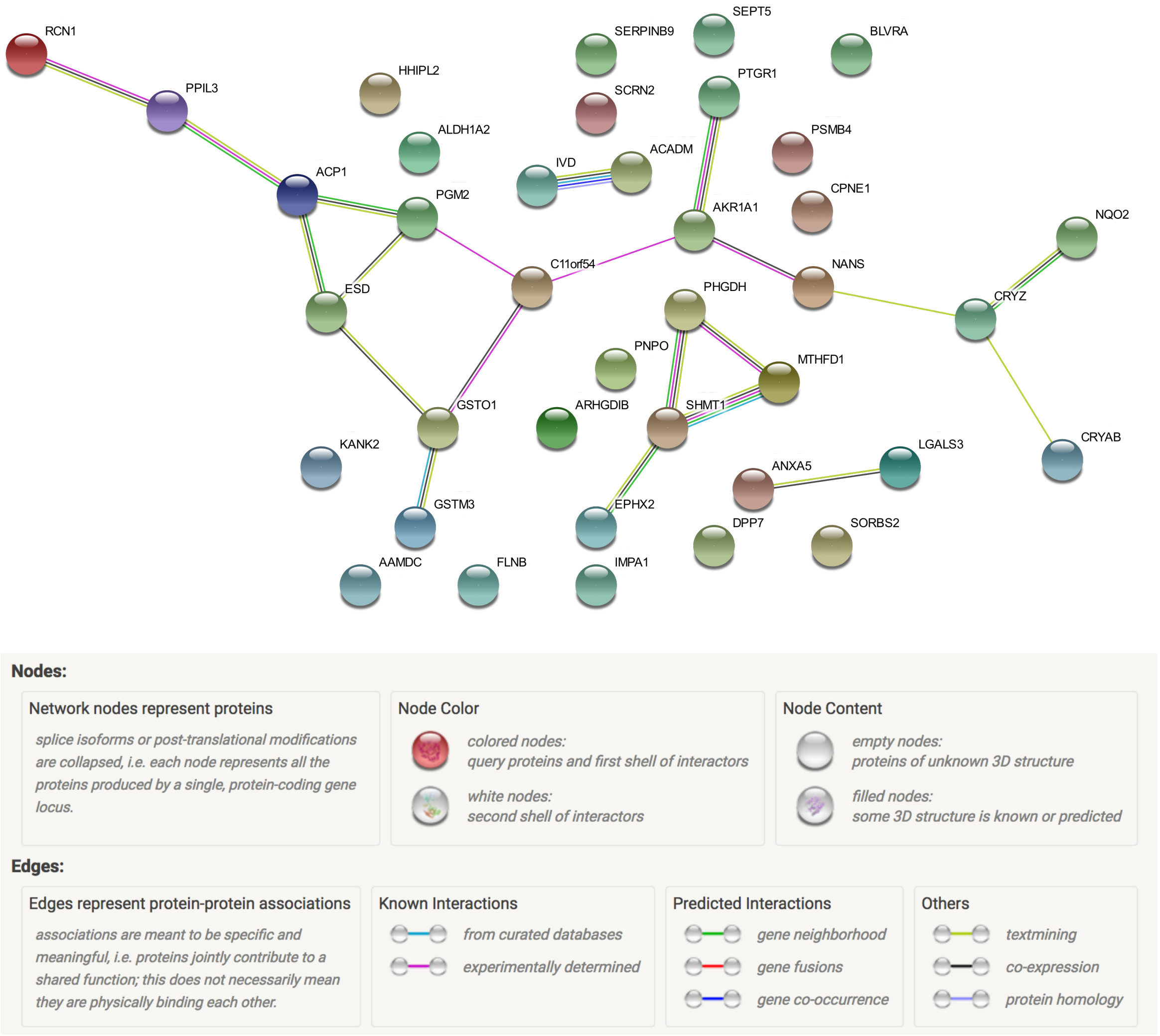

Supplementary Figure 4. Protein-protein interaction network for 38 proteins with pQTLs detected in cartilage (STRING v11.0).

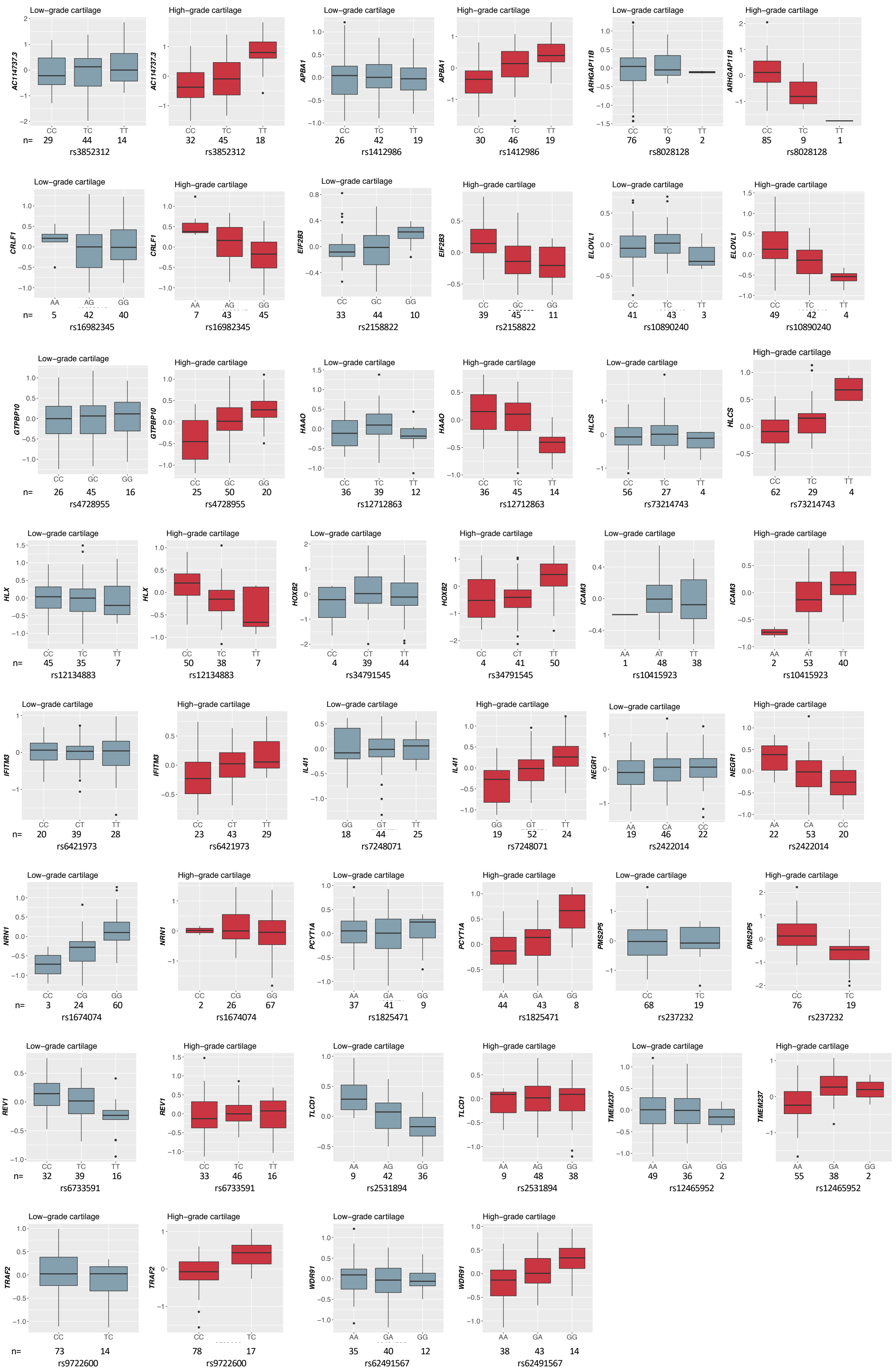
Low−grade cartilage AC114737.3 CC TC TT n= 29 44 14 rs3852312
High−grade cartilage AC114737.3 CC TC TT 32 45 18 rs3852312
Low−grade cartilage APBA1 CC TC TT 26 42 19 rs1412986
High−grade cartilage APBA1 CC TC TT 30 46 19 rs1412986
Low−grade cartilage ARHGAP11B CC TC TT 76 9 2 rs8028128
High−grade cartilage ARHGAP11B CC TC TT 85 9 1 rs8028128
Low−grade cartilage CRLF1 AA AG GG n= 5 42 40 rs16982345
High−grade cartilage CRLF1 AA AG GG 7 43 45 rs16982345
Low−grade cartilage EIF2B3 CC GC GG 33 44 10 rs2158822
High−grade cartilage EIF2B3 CC GC GG 39 45 11 rs2158822
Low−grade cartilage ELOVL1 CC TC TT 41 43 3 rs10890240
High−grade cartilage ELOVL1 CC TC TT 49 42 4 rs10890240
Low−grade cartilage GTPBP10 CC GC GG n= 26 45 16 rs4728955
High−grade cartilage GTPBP10 CC GC GG 25 50 20 rs4728955
Low−grade cartilage HAAO CC TC TT 36 39 12 rs12712863
High−grade cartilage HAAO CC TC TT 36 45 14 rs12712863
Low−grade cartilage HLCS CC TC TT 56 27 4 rs73214743
High−grade cartilage HLCS CC TC TT 62 29 4 rs73214743
Low−grade cartilage HLX CC TC TT n= 45 35 7 rs12134883
High−grade cartilage HLX CC TC TT 50 38 7 rs12134883
Low−grade cartilage HOXB2 CC CT TT 4 39 44 rs34791545
High−grade cartilage HOXB2 CC CT TT 4 41 50 rs34791545
Low−grade cartilage ICAM3 AA AT TT 1 48 38 rs10415923
High−grade cartilage ICAM3 AA AT TT 2 53 40 rs10415923
Low−grade cartilage IFITM3 CC CT TT n= 20 39 28 rs6421973
High−grade cartilage IFITM3 CC CT TT 23 43 29 rs6421973
Low−grade cartilage IL4I1 GG GT TT 18 44 25 rs7248071
High−grade cartilage IL4I1 GG GT TT 19 52 24 rs7248071
Low−grade cartilage NEGR1 AA CA CC 19 46 22 rs2422014
High−grade cartilage NEGR1 AA CA CC 22 53 20 rs2422014
Low−grade cartilage NRN1 CC CG GG n= 3 24 60 rs1674074
High−grade cartilage NRN1 CC CG GG 2 26 67 rs1674074
Low−grade cartilage PCYT1A AA GA GG 37 41 9 rs1825471
High−grade cartilage PCYT1A AA GA GG 44 43 8 rs1825471
Low−grade cartilage PMS2P5 CC TC 68 19 rs237232
High−grade cartilage PMS2P5 CC TC 76 19 rs237232
Low−grade cartilage REV1 CC TC TT n= 32 39 16 rs6733591
High−grade cartilage REV1 CC TC TT 33 46 16 rs6733591
Low−grade cartilage TLCD1 AA AG GG 9 42 36 rs2531894
High−grade cartilage TLCD1 AA AG GG 9 48 38 rs2531894
Low−grade cartilage TMEM237 AA GA GG 49 36 2 rs12465952
High−grade cartilage TMEM237 AA GA GG 55 38 2 rs12465952
Low−grade cartilage TRAF2 CC TC n= 73 14 rs9722600
High−grade cartilage TRAF2 CC TC 78 17 rs9722600
Low−grade cartilage WDR91 AA GA GG 35 40 12 rs62491567
High−grade cartilage WDR91 AA GA GG 38 43 14 rs62491567

Supplementary Figure 5. Genes with molecular QTL present in high-grade, but not low-grade cartilage (posterior probability m>0.9 and m<0.1, respectively).

For each of the 23 genes, the variant with strongest differential eQTL signal is shown. The boxplots show expression (residuals after regressing the normalised expression data on the 15 PEER factors, sex and array) at 25th to 75th percentiles, with centre at the median and whiskers extend to 1.5 times the interquartile range. n: number of individuals included in the analysis for each genotype.

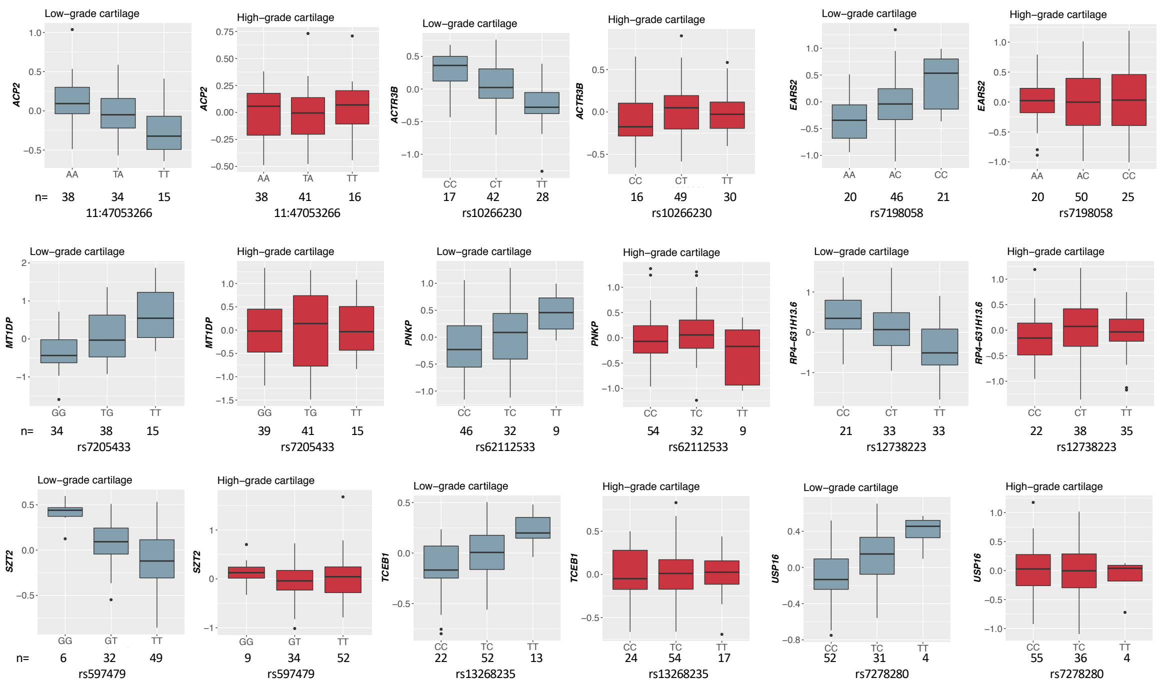

Supplementary Figure 6. Genes with molecular QTL present in low-grade, but not high-grade cartilage (posterior probability m>0.9 and m<0.1, respectively).

For each of the 9 genes, the variant with strongest differential eQTL signal is shown. The boxplots show expression (residuals after regressing the normalised expression data on the 15 PEER factors, sex and array) at 25th to 75th percentiles, with centre at the median and whiskers extend to 1.5 times the interquartile range. n: number of individuals included in the analysis for each genotype.

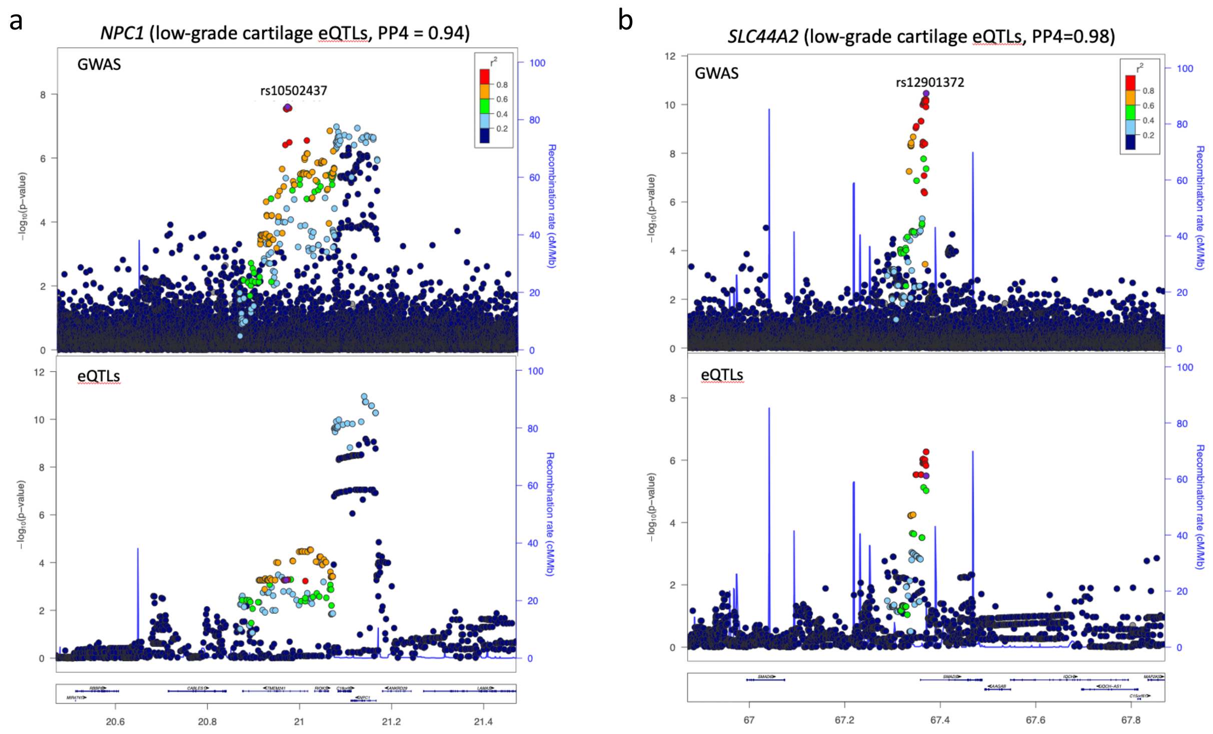

Supplementary Figure 7. Co-localisation of osteoarthritis GWAS signals and low-grade cartilage *cis*-eQTLs for *NPC1* and *SMAD3*.

The plots show GWAS and low-grade cartilage eQTL p-values for regions surrounding

a, GWAS signal with index variant rs10502437 and *NCP1* eQTLs in low-grade cartilage

b, GWAS signal with index variant rs12901372 and SLC44A2 eQTLs in low-grade cartilage.

For both GWAS signals, we observed colocalisation with eQTLs in both low-grade and high-grade cartilage, and the plots for high-grade cartilage are shown in Fig. 3. Each plot shows 1Mb region centered around the GWAS index SNP (purple); each point represents a genetic variant. Top panels show GWAS p-values, bottom panels QTL p-values for the indicated gene. Here and in Fig. 3, LD between variants was calculated using UK Biobank. PP4: posterior probability for colocalisation.

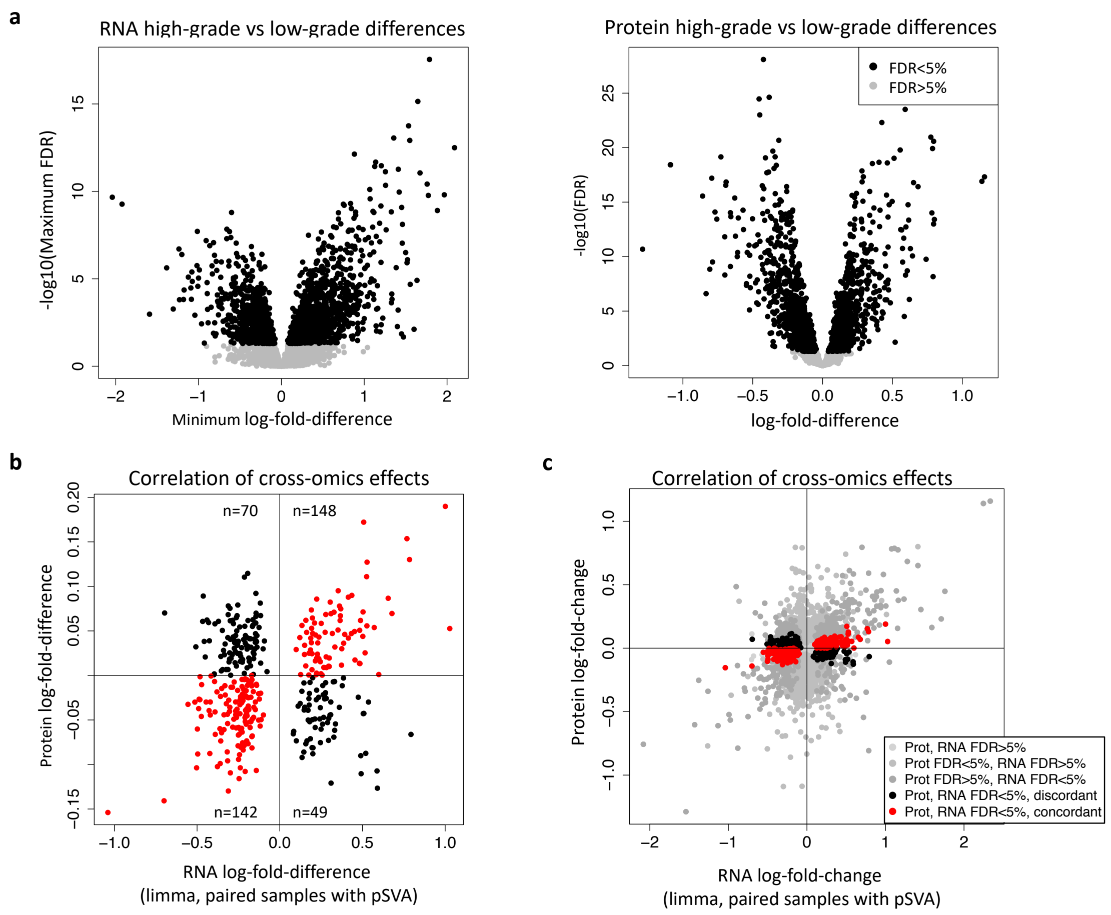

Supplementary Figure 8. Molecular differences between high-grade and low-grade cartilage.

- **a,** Wide-spread RNA-level (left) and protein-level (right) differences between high-grade and low-grade cartilage. The RNA plot shows conservative results based on different approaches (see Methods), with 2,557 differentially expressed genes significant at 5% FDR in all approaches.
- **b,** RNA- and protein-level log-fold differences for 409 genes with significant cross-omics differences between high-grade and low-grade cartilage (see panel C for all genes). The direction of difference agrees for 290 of the 409 genes (71%; binomial $P<1.0\times10^{-17}$), with a strong correlation of effect sizes (Pearson r=0.63, $P<10^{-10}$).

c, RNA- and protein-level log-fold-differences for all genes measured on both omics levels. The x-axis shows gene expression differences between low-grade and high-grade cartilage as quantified by limma in an analysis including the technical covariates as identified by pSVA as well as pairing samples from the same patients. The genes highlighted black or red were significant on both RNA- and protein-level, see also (B). The correlation of effect sizes across all genes was also significant (Pearson r=0.32, P<10^{-10}).

a

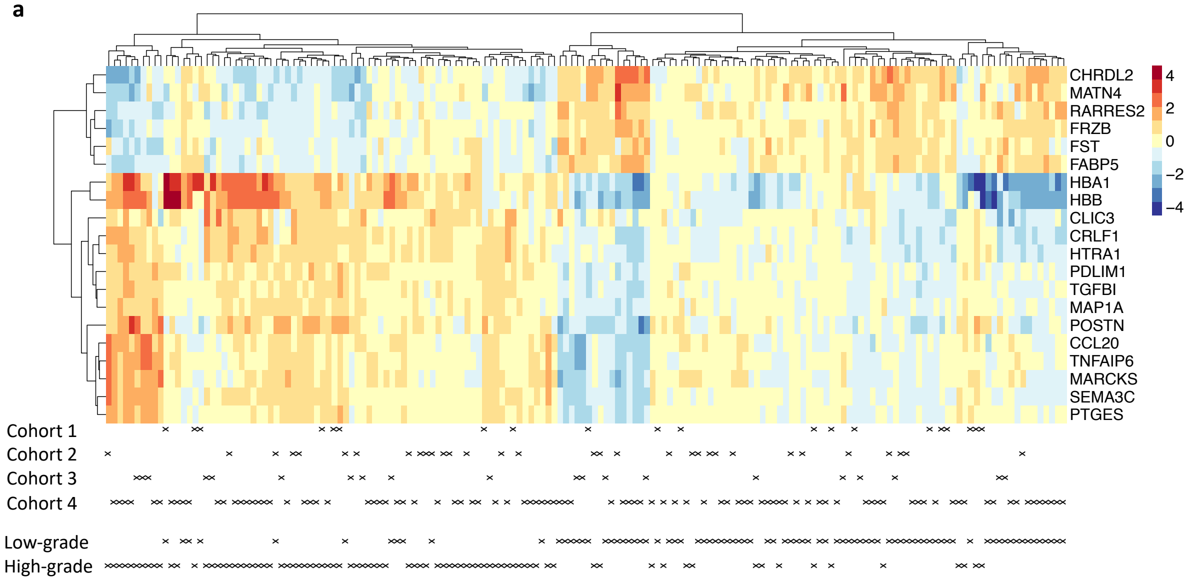

b

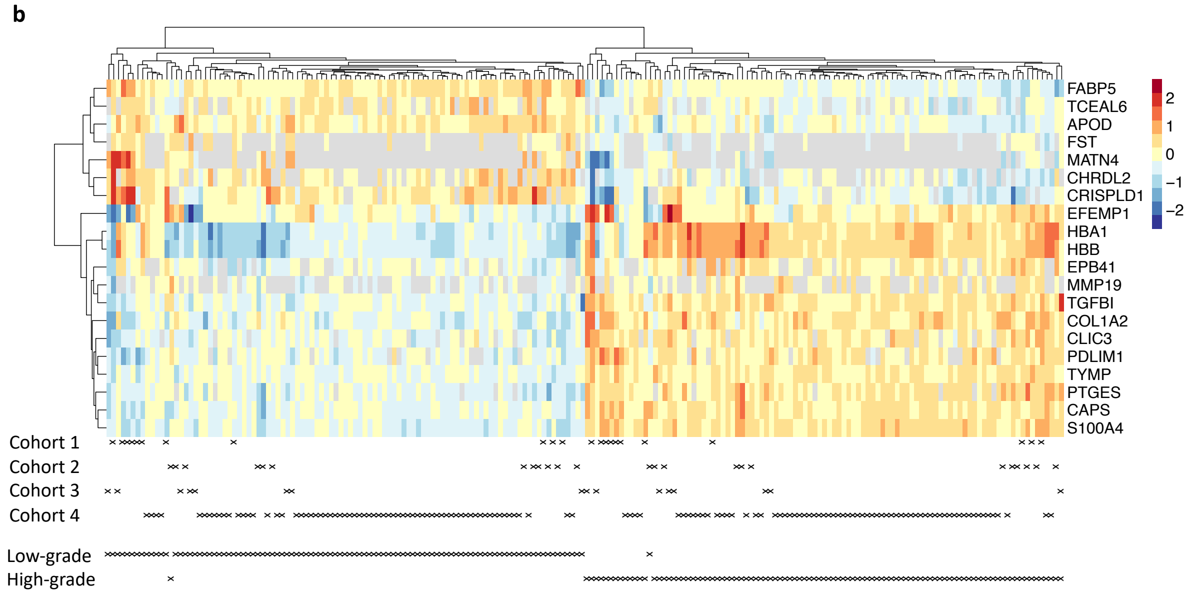

Supplementary Figure 9. Heatmap of gene expression (a) and protein abundance (b) for top 20 genes respectively proteins with highest absolute log-fold-differences between high-grade and low-grade cartilage, among all genes with cross-omics significant and concordant differences.

a, Gene expression residuals after regressing out patient-level effects and 10 additional covariates accounting for technical variation (as identified by SVAseq and used in differential gene expression analysis). The 20 genes were selected based on log-fold-

differences quantified by limma from the paired-sample analysis including 10 technical covariates.

b, Protein abundance residuals after regressing out patient-level effects from quantile normalised data used for differential abundance analysis. Grey colour indicates proteins not quantified in a given sample.

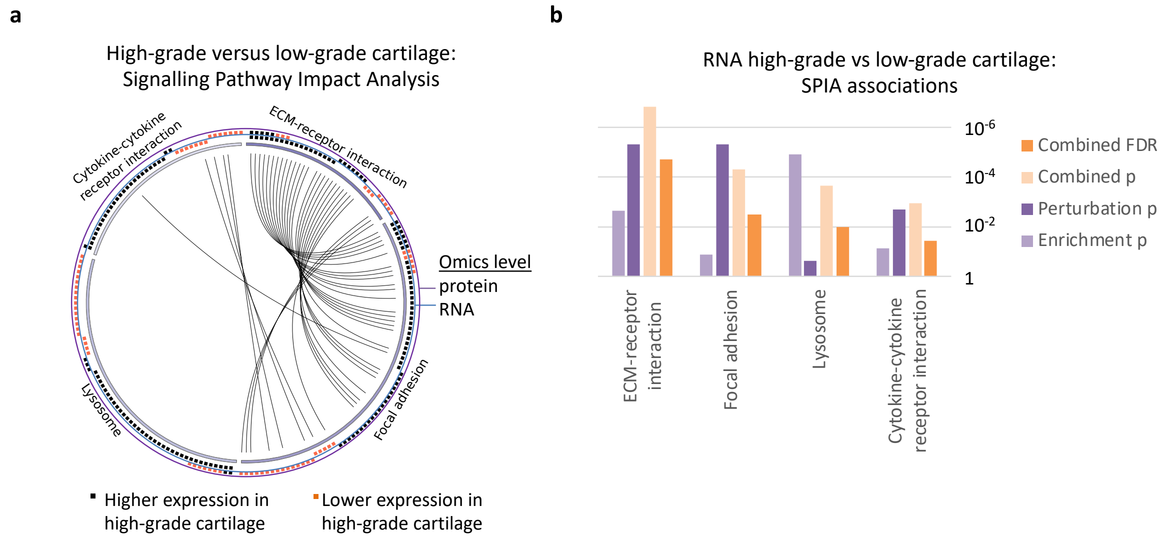

Supplementary Figure 10. Biological pathways and processed associated with molecular differences between high-grade and low-grade cartilage.

a, Signalling Pathway Impact Analysis (SPIA) identified biological pathways associated with differences between high-grade and low-grade cartilage. Pathways with significant results at 5% FDR based on RNA-level changes are shown, all activated in high-grade cartilage. Boxes on the outside circles represent individual genes, with arches connecting the same gene across pathways.

b, Signalling Pathway Impact Analysis (SPIA) p-values for pathways that show significant association with differences between high-grade and low-grade cartilage based on RNA-level changes. Enrichment p: p-value from over-representation analysis of genes; Perturbation p: p-value for perturbation of the pathway based on gene log-fold-differences; Combined p: p-value from combining enrichment and perturbation p-values. Pathways with significant results at 5% FDR based on RNA-level changes are shown. See also full results in Supplementary Data 3.

Data available online

Raw data: uploaded to EGA/PRIDE (accession numbers see Data availability statement)

1. RNA sequencing data [EGA]
2. Proteomics data [PRIDE]
3. Genotype data [EGA]

Data uploaded to https://hmgubox.helmholtz-muenchen.de/d/fc1fcf65a6724152b7f9/

Significant molQTLs

4. Significant *cis*-eQTLs for high-grade and low-grade cartilage
5. Significant *cis*-pQTLs for high-grade and low-grade cartilage

TSS data

6. TSS for each gene

Full colocalisation results (probabilities for each locus, tissue, and omics level)

7. Colocolisation: full results

Supplementary References

1. Robins, C., *et al.* Genetic control of the human brain proteome. *bioRxiv*, 816652 (2019).
2. Szklarczyk, D., *et al.* STRING v11: protein-protein association networks with increased coverage, supporting functional discovery in genome-wide experimental datasets. *Nucleic Acids Res* **47**, D607-D613 (2019).
3. Gregori, J., *et al.* Batch effects correction improves the sensitivity of significance tests in spectral counting-based comparative discovery proteomics. *J. Proteom.* **75**, 3938-3951 (2012).
4. Kuligowski, J., *et al.* Detection of batch effects in liquid chromatography-mass spectrometry metabolomic data using guided principal component analysis. *Talanta* **130**, 442-448 (2014).
5. Fortelny, N., Overall, C.M., Pavlidis, P. & Freue, G.V.C. Can we predict protein from mRNA levels? *Nature* **547**, E19-e20 (2017).
6. Nagao, M., *et al.* Vascular endothelial growth factor in cartilage development and osteoarthritis. *Sci. Rep.* **7**, 13027 (2017).
7. Takeshita, N., *et al.* Alleviating effects of AS1892802, a rho kinase inhibitor, on osteoarthritic disorders in rodents. *J. Pharmacol. Sci.* **115**, 481-489 (2011).
