# Supplementary material for: A molecular quantitative trait locus map for osteoarthritis
Source: Nat Commun. 2021 Feb 26;12:1309. doi: 10.1038/s41467-021-21593-7 (PMC7910531; doi:10.1038/s41467-021-21593-7)
Supplement: Supplementary file 3 — Reporting Summary [file 41467_2021_21593_MOESM3_ESM.pdf]

nature research

Corresponding author(s): Eleftheria Zeggini and J Mark Wilkinson

Last updated by author(s): Jan 23, 2021

Reporting Summary

Nature Research wishes to improve the reproducibility of the work that we publish. This form provides structure for consistency and transparency in reporting. For further information on Nature Research policies, see our Editorial Policies and the Editorial Policy Checklist.

Statistics

For all statistical analyses, confirm that the following items are present in the figure legend, table legend, main text, or Methods section.

n/a	Confirmed	
☐	☒	The exact sample size (*n*) for each experimental group/condition, given as a discrete number and unit of measurement
☐	☒	A statement on whether measurements were taken from distinct samples or whether the same sample was measured repeatedly
☐	☒	The statistical test(s) used AND whether they are one- or two-sided<br>*Only common tests should be described solely by name; describe more complex techniques in the Methods section.*
☐	☒	A description of all covariates tested
☐	☒	A description of any assumptions or corrections, such as tests of normality and adjustment for multiple comparisons
☒	☐	A full description of the statistical parameters including central tendency (e.g. means) or other basic estimates (e.g. regression coefficient) AND variation (e.g. standard deviation) or associated estimates of uncertainty (e.g. confidence intervals)
☐	☒	For null hypothesis testing, the test statistic (e.g. *F*, *t*, *r*) with confidence intervals, effect sizes, degrees of freedom and *P* value noted<br>*Give P values as exact values whenever suitable.*
☒	☐	For Bayesian analysis, information on the choice of priors and Markov chain Monte Carlo settings
☒	☐	For hierarchical and complex designs, identification of the appropriate level for tests and full reporting of outcomes
☐	☒	Estimates of effect sizes (e.g. Cohen's *d*, Pearson's *r*), indicating how they were calculated

Our web collection on statistics for biologists contains articles on many of the points above.

Software and code

Policy information about availability of computer code

Data collection	samtools v1.3.1<br>biobambam v0.0.191<br>FastQC v0.11.5<br>salmon v0.8.2<br>tximport v1.4.0<br>Proteome Discoverer v2.1<br>GenCall (Illumina)<br>Genotype mapping to GRC37/hg19 on-line tool (http://www.well.ox.ac.uk/~wrayner/strand/index.html)<br>Genotype HRC preparation checking tool v4.2.7 (http://www.well.ox.ac.uk/~wrayner/tools/)<br>Genotype post-imputation data checking program v1.0.2 (http://www.well.ox.ac.uk/~wrayner/tools/Post-Imputation.html)<br>Eagle2
Data analysis	R v3.4.0<br>Plink v1.9<br>PEER v1.0<br>GTEx modified version of FastQTL (https://github.com/francois-a/fastqtl; v6p)<br>coloc-fast (https://github.com/tobyjohnson/gtx/blob/526120435bb3e29c39fc71604eee03a371ec3753/R/coloc.R)<br>Meta-Tissue v0.5<br>Ensembl Variant Effect Predictor (http://grch37.ensembl.org/Homo_sapiens/Tools/VEP/)<br>STRING v11.0, https://string-db.org/<br>limma v3.32.10<br>edgeR v3.18.1

DESeq2 v1.16.1
SVA v3.24.4
pheatmap v1.0.12
MAGMA v1.06
SPIA v2.28.0
GOseq v1.28.0
ConnectivityMap (https://clue.io, accessed 03 March 2019)

For manuscripts utilizing custom algorithms or software that are central to the research but not yet described in published literature, software must be made available to editors and reviewers. We strongly encourage code deposition in a community repository (e.g. GitHub). See the Nature Research guidelines for submitting code & software for further information.

Data

Policy information about availability of data

All manuscripts must include a data availability statement. This statement should provide the following information, where applicable:

- Accession codes, unique identifiers, or web links for publicly available datasets
- A list of figures that have associated raw data
- A description of any restrictions on data availability

The RNA sequencing data have been deposited to EGA (accession IDs EGAD00001005215, EGAD00001003355, EGAD00001003354, EGAD00001001331).
The proteomics data reported in this paper have been deposited to the PRIDE archive (accession IDs PXD014666, PXD006673, PXD002014).
The genotype data have also been deposited to EGA (accession IDs EGAD00010001746, EGAD00010001285, EGAD00010001292, EGAD00010000722).

Data from the 1000 Genomes Project are publicly available (https://www.internationalgenome.org). We also used publicly available data on osteoarthritis differential gene expression from the RAAK Study (https://git.lumc.nl/rcoutinhodealmeida/miRNAmRNA, accessed 20 June 2020).

Further data including the TSS information, all significant molQTLs, and full colocalisation results can be obtained online from https://hmgubox.helmholtz-muenchen.de/d/fc1fcf65a6724152b7f9/. The full molecular QTL data and molecular differences between high-grade and low-grade cartilage are available through the Downloads page of the Musculoskelatal Knowledge Portal (mskkp.org).

Field-specific reporting

Please select the one below that is the best fit for your research. If you are not sure, read the appropriate sections before making your selection.

☒ Life sciences ☐ Behavioural & social sciences ☐ Ecological, evolutionary & environmental sciences

For a reference copy of the document with all sections, see nature.com/documents/nr-reporting-summary-flat.pdf

Life sciences study design

All studies must disclose on these points even when the disclosure is negative.

Sample size	Sample size was determined by the availability of the human samples.
Data exclusions	We excluded samples that failed quality control, or samples from individuals of non-European ancestry. The number of samples excluded is listed below and shown in in Supplementary Data 7. All exclusion criteria were determined before any analyses were conducted.<br>RNA data: we excluded 9 samples due to FastQC quality checks, 4 samples due to low mapping rate (<80%), 3 samples due to non-European ancestry recorded in the clinic, 18 samples due to low RIN (<5), 2 samples as duplicates, 8 samples due to abnormal gene read density plots, 8 samples due to non-European ancestry determined from genotype data.<br>Protein data: we excluded 2 samples due to non-European ancestry recorded in the clinic, and 6 samples due to non-European ancestry determined from genotype data.<br>Genotype data: we excluded 7 samples as duplicates, 3 samples due to non-European ancestry determined from genotype data, and 2 samples due to absence of RNA and protein data.
Replication	As no genome-wide molecular QTL data for cartilage and synovium were available before this study, a replication analysis of the molecular QTLs was not possible.<br>To replicate gene expression and protein abundance differences between high-grade and low-grade cartilage, we used an independent, previously published analysis of RNA sequencing data from 35 osteoarthritis patients. As described in the paper, of the differentially expressed genes and proteins in our discovery analysis, 65.9% and 68.3% showed a concordant direction of effect in the replication data, respectively (both Fisher's p<10-10, Supplementary Data 4). We found significantly higher concordance where replication power was highest (88.5% for genes with cross-omics higher expression in high-grade cartilage, compared to 66.7% for genes with cross-omics lower expression in high-grade cartilage, Fisher's p=8.6x10-6, Supplementary Data 4).
Randomization	not applicable due to no experimental treatments
Blinding	All samples in this study were collected from osteoarthritis patients. Samples from each tissue were analysed separately to identify molecular QTL analysis, so blinding was not applicable. In the analysis of gene expression and protein abundance differences between high-grade and low-grade cartilage, tissue type was the primary factor of interest, so blinding was not applicable.

Reporting for specific materials, systems and methods

We require information from authors about some types of materials, experimental systems and methods used in many studies. Here, indicate whether each material, system or method listed is relevant to your study. If you are not sure if a list item applies to your research, read the appropriate section before selecting a response.

Materials & experimental systems

n/a	Involved in the study
☒	☐ Antibodies
☒	☐ Eukaryotic cell lines
☒	☐ Palaeontology and archaeology
☒	☐ Animals and other organisms
☐	☒ Human research participants
☒	☐ Clinical data
☒	☐ Dual use research of concern

Methods

n/a	Involved in the study
☒	☐ ChIP-seq
☒	☐ Flow cytometry
☒	☐ MRI-based neuroimaging

Human research participants

Policy information about studies involving human research participants

Population characteristics	We collected tissue samples from 115 patients undergoing total joint replacement surgery for osteoarthritis: 12 knee osteoarthritis patients (cohort 1; 2 women, 10 men, age 50-88 years, mean 68 years); 20 knee osteoarthritis patients (cohort 2; 14 women, 6 men, age 54-82 years, mean 70 years); 13 hip osteoarthritis patients (cohort 3; 8 women, 5 men, age 44-84 years, mean 62 years); 70 knee osteoarthritis patients (cohort 4; 42 women, 28 men, age 38-84 years, mean 70 years).
Recruitment	Participants comprised men and women with known knee or hip osteoarthritis undergoing joint replacement for symptomatic disease. We aimed to recruit participants of European ancestry (later confirmed by genotype analysis), so our findings cannot be generalized to dissimilar populations. For knee osteoarthritis patients, the selection criteria also included absence of inflammatory arthropathy, drugs known to affect bone or cartilage metabolism or disease modifying agents for inflammatory disorders. Participation in the study was not associated with any actual or perceived secondary health gains.
Ethics oversight	This work was approved by Oxford NHS REC C (10/H0606/20 and 15/SC/0132). Samples were collected under Human Tissue Authority license 12182, Sheffield Musculoskeletal Biobank, University of Sheffield, UK; and under National Research Ethics approval reference 11/EE/0011, Cambridge Biomedical Research Centre Human Research Tissue Bank, Cambridge University Hospitals, UK. All patients provided written, informed consent prior to participation in the study.

Note that full information on the approval of the study protocol must also be provided in the manuscript.
