# Supplementary material for: A molecular quantitative trait locus map for osteoarthritis
Source: Nat Commun. 2021 Feb 26;12:1309. doi: 10.1038/s41467-021-21593-7 (PMC7910531; doi:10.1038/s41467-021-21593-7)
Supplement: Supplementary file 4 — Description of Additional Supplementary Files [file 41467_2021_21593_MOESM4_ESM.pdf]

Description of Additional Supplementary Files

Supplementary Data 1. Detailed list of differential eQTLs, i.e. variants with high posterior probability for presence of eQTL effect in high-grade cartilage (m>0.9) and low for presence in low-grade cartilage (m<0.1), or vice versa.

Supplementary Data 2. Genes with significant cross-omics differences between high-grade and low-grade cartilage.

Supplementary Data 3. Pathways and gene sets associated with significant RNA-level and/or protein-level differences between high-grade and low-grade cartilage.

Supplementary Data 4. Replication of molecular differences between high-grade and low-grade cartilage, based on an independent RNA sequencing dataset of 35 patients.

Supplementary Data 5. Genes with significantly different expression profiles between high-and low-grade cartilage that were also found to be associated with genetic risk of osteoarthritis in a recent GWAS (multiple-testing corrected significance threshold of $P<1.02 \times 10^{-6}$).

Supplementary Data 6. Comparison of perturbations by compounds, gene knockdown or overexpression, to differences between high-grade and low-grade cartilage.
The comparison was based on data from ConnectivityMap and genes with higher expression in high-grade than in low-grade cartilage on both RNA- and protein-level, see Methods.

Supplementary Data 7. List of all assayed patient tissue samples with detailed information including cohort, batch, and quality control exclusions.
