# Supplementary material for: A molecular quantitative trait locus map for osteoarthritis
Source: Nat Commun. 2021 Feb 26;12:1309. doi: 10.1038/s41467-021-21593-7 (PMC7910531; doi:10.1038/s41467-021-21593-7)
Supplement: Supplementary file 2 — Peer Review File [file 41467_2021_21593_MOESM2_ESM.pdf]

Reviewer #1 (Remarks to the Author):

The manuscript of Steinberg et al has improved a lot and represents a valuable contribution to the literature. The paper reads much better and has improved in focus and flow.

My main remaining concern is the interpretation of the differential gene expression analysis comparing low versus high grade analysis. This analysis revealed a total of 2557 genes differentially expressed (approximately 50% of the total amount of genes that were expressed in cartilage). The authors then use this information as a further argument to identify functional effector genes and/or likely drug-compounds. Given the high amount of genes differentially expressed, it is likely that aspecific genes are identified, which makes this part of the manuscript highly speculative.
o Page 6, last paragraph states: "91 of the genes with significantly expressed profiles between high and low grade cartilage (54 with concordant direction of effect in the independent replication dataset) were also associated with genetic risk for osteoarthritis?"
◊ Only 54 of the 91 diff. expressed genes replicated in the same direction
◊ How many genes were studied in total, and how many were differentially expressed?

Reviewer #2 (Remarks to the Author):

All my comments have been addressed. My major concerns were from the mouse models and the stratification of OA patients, which have both now been removed. This still leaves an important analysis of molecular traits in relevant tissues for OA in a large data set.

Reviewer #3 (Remarks to the Author):

In their study, Steinberg et al. analyzed an impressive set of data comprising genetic, gene expression and protein data from cartilage and synovium of patients with osteoarthritis. Using this data they identified eQTLs in cartilage (high- and low-grade) and synovium, pQTLs in cartilage (high- and low-grade), they analyzed changes in gene and protein expression between low- and high grade cartilage and they applied an algorithm to identify therapeutics with a potential use in the treatment of osteoarthritis. The eQTL/pQTL parts of the analysis are novel and of particular interest. The comparison of gene and protein expression between high and low grade cartilage is solid and profits from the high number of samples.

The organization of the manuscript and the presentation of the data make it difficult to appreciate the value and significance of the results. The study seems to be split between the genetic part (molQTLs) and the comparison between low- and high-grade cartilage. The two parts do not really complement each other and the results of each part could be taken further to make the conclusions more interesting. In its present form, the study does not follow or build a hypothesis that is then tested and leads to a conclusion. It appears rather like a compilation of various analyses.

Specific comments:

1) The manuscript would benefit from subheadings and text subdivisions. In particular, a thorough discussion of the data is missing.
2) The presentation of the data could be improved by re-arrangement and addition of figures. For example, Figure 1 could contain a) Ext. Data Fig.1a, b) Ext. Data Fig.2a, c) Fig. 1a, d) Fig. 1b. It would also be of interest to show some examples of pQTLs. Extended Data Fig. 4 is not really readable in the current form.
3) It would be interesting to learn more about the eQTLs and pQTLs found. The top eQTLs and the pQTLs should be presented in a table or suppl. table. Were some of the pQTLs also eQTLs? Maybe a

pathway enrichment analysis could be done for the eQTLs and a protein-protein interaction network for the pQTLs.
4) Differential eQTLs were identified between low- and high grade cartilage, but not between cartilage and synovium. A major asset of this study is the inclusion of several disease-relevant tissues (synovium and cartilage), but this data is not really presented in the paper. It is remarkable that almost a fifth of the eQTLs in OA specifically occur in the synovium, but not in the cartilage. It would be of high interest to know which ones they are, what their function is and whether these could be therapeutic targets for OA.
5) The differential eQTLs between high- and low grade cartilage are of interest, but also here the reader is a bit left alone with the results. Which pathways were enriched in low-grade cartilage, which in high-grade cartilage? The relationship between high and low grade cartilage should be discussed to help the reader to interpret the results. Is this considered as two different end-stage degrees of the disease or is low grade cartilage considered as precursor of high grade cartilage?
6) For the changes in gene/protein expression part of the study, maybe a heatmap showing the most differentially expressed genes/proteins could be shown as Figure. It would be interesting to learn more on the expected effect of the differential genes and pathways (e.g. lysosome, cytokine-cytokine receptor interactions). Furthermore, the effect of changed expression of some of the less known proteins found to be different between the two stages (e.g. the aldehyde dehydrogenases) could be elucidated with functional experiments, but this is probably out of the scope of the study.

Caroline Ospelt

<u>Response to reviewers' comments</u>

Reviewer #1

The manuscript of Steinberg et al has improved a lot and represents a valuable contribution to the literature. The paper reads much better and has improved in focus and flow.

We thank the reviewer for their feedback and for helping improve the paper.

My main remaining concern is the interpretation of the differential gene expression analysis comparing low versus high grade analysis. This analysis revealed a total of 2557 genes differentially expressed (approximately 50% of the total amount of genes that were expressed in cartilage). The authors then use this information as a further argument to identify functional effector genes and/or likely drug-compounds. Given the high amount of genes differentially expressed, it is likely that aspecific genes are identified, which makes this part of the manuscript highly speculative.

We found that 2,557 of 15,249 genes quantified in the RNA data were significantly differentially expressed (<17%). These data provide a characterisation of the wide-spread molecular changes of cartilage degradation, and it is indeed not expected that all these genes are causal. However, even genes that might not be causal in the degradation could still present valuable drug targets, for example, if located up-stream or down-stream in the key molecular processes.

Moreover, we use the molecular signatures as a basis for the identification of candidate therapeutic compounds. Specifically, we sought compounds that can reverse the signature by reducing the expression of genes with cross-omics higher expression in high-grade cartilage. This uses information across the broader molecular signature of cartilage degradation and does not rely on changes in individual genes being causal. Encouragingly, several of the top shortlisted compounds have known biological relevance to osteoarthritis, supporting the strength of this approach.

We agree that future biological experiments (e.g. by gene knock-out or over-expression) will be key to prove which gene expression changes are causal in disease development and progression. The genes with convergent evidence from this study as well as osteoarthritis GWAS are presented to help such research and accelerate the pathway to translation. We have added a corresponding section to the Discussion to emphasise this final point.

o Page 6, last paragraph states: "91 of the genes with significantly expressed profiles between high and low grade cartilage (54 with concordant direction of effect in the independent replication dataset) were also associated with genetic risk for osteoarthritis?"
Only 54 of the 91 diff. expressed genes replicated in the same direction
How many genes were studied in total, and how many were differentially expressed?
We note that the replication dataset was substantially smaller (35 patients only), which would reduce the power to detect and replicate true positive associations.

In the analysis in question, we considered the 238 genes with significant gene-level association with osteoarthritis in a recent GWAS, and which were also assayed on at least one omics level in the current study, as described in the Methods section:
"From the recent UK Biobank and arcOGEN GWAS meta-analysis2, we obtained the results of a gene-level analysis for each of the four osteoarthritis phenotypes (self-reported plus hospital diagnosed, hospital diagnosed knee or hip, hospital diagnosed knee, hospital diagnosed hip), as described in the GWAS paper. … After accounting for the effective number of tests across phenotypes and genes using a Bonferroni correction, 320 of 18,449

genes showed significant association with at least one phenotype. Of these genes, 238 genes were compared between low-grade and high-grade cartilage on at least one omics level and had uniquely corresponding Ensembl gene ID and gene name."

We have added ***further details to clarify in the Results*** *(underlined text):*
"91 of the genes with significantly different expression profiles between high- and low-grade cartilage were also associated with genetic risk of osteoarthritis (i.e. among the 238 genes with gene-level significant association in a recent meta-analysis[8]; Supplementary Table 5). 54 of these genes also showed concordant molecular differences in the independent replication dataset, providing further evidence for the potential involvement of these genes in osteoarthritis disease processes."

Information from additional studies would be required to determine whether an absence of clear association in the replication dataset for the remaining genes is due to limited power or a true lack of association. As described in the Results, when considering all genes, "We found significantly higher concordance where replication power was highest (88.5% for genes with cross-omics higher expression in high-grade cartilage, compared to 66.7% for genes with cross-omics lower expression in high-grade cartilage, Fisher's p=8.6x10-6, Supplementary Table 4)."

Reviewer #2

All my comments have been addressed. My major concerns were from the mouse models and the stratification of OA patients, which have both now been removed. This still leaves an important analysis of molecular traits in relevant tissues for OA in a large data set.

We thank the reviewer for their help in improving the paper.

Reviewer #3

In their study, Steinberg et al. analyzed an impressive set of data comprising genetic, gene expression and protein data from cartilage and synovium of patients with osteoarthritis. Using this data they identified eQTLs in cartilage (high- and low-grade) and synovium, pQTLs in cartilage (high- and low-grade), they analyzed changes in gene and protein expression between low- and high grade cartilage and they applied an algorithm to identify therapeutics with a potential use in the treatment of osteoarthritis. The eQTL/pQTL parts of the analysis are novel and of particular interest. The comparison of gene and protein expression between high and low grade cartilage is solid and profits from the high number of samples.

The organization of the manuscript and the presentation of the data make it difficult to appreciate the value and significance of the results. The study seems to be split between the genetic part (molQTLs) and the comparison between low- and high-grade cartilage. The two parts do not really complement each other and the results of each part could be taken further to make the conclusions more interesting. In its present form, the study does not follow or build a hypothesis that is then tested and leads to a conclusion. It appears rather like a compilation of various analyses.

Specific comments:

1) The manuscript would benefit from subheadings and text subdivisions. In particular, a thorough discussion of the data is missing.

We thank the reviewer for the suggestion and have included subheadings and text subdivisions, as well as a separate discussion section.

2) The presentation of the data could be improved by re-arrangement and addition of figures. For example, Figure 1 could contain a) Ext. Data Fig.1a, b) Ext. Data Fig.2a, c) Fig. 1a, d) Fig. 1b. It would also be of interest to show some examples of pQTLs. Extended Data Fig. 4 is not really readable in the current form.

We have incorporated all of these suggestions in the revised version:

First, we have ***reorganised the main display items****, which now are:*

- *Figure 1: Study approach (was Ext. Data Fig 1a)*
- *Figure 2: cis-eQTLs in osteoarthritis disease tissue (was Ext. Data Fig 2a, Fig 1a-b)*
- *Figure 3: GWAS and molecular QTL P-values in regions with co-localisation of the associations (was Fig 2)*

Second, we now also show example pQTL effects in ***Supplementary Fig. 3.***

Finally, we have improved ***Supplementary Fig. 5*** *(was Extended Data Fig. 4) to make the text readable.*

3) It would be interesting to learn more about the eQTLs and pQTLs found. The top eQTLs and the pQTLs should be presented in a table or suppl. table. Were some of the pQTLs also eQTLs? Maybe a pathway enrichment analysis could be done for the eQTLs and a protein-protein interaction network for the pQTLs.

We thank the reviewer for these suggestions.

The full list of differential eQTLs is presented in ***Supplementary Table 1****.*

We want to make all molecular QTLs from this study available to the broader community upon publication, and are doing so through two approaches designed to suit different purposes:

1. *To enable future large-scale computational analyses, text files with all significant eQTLs and pQTLs have been uploaded to a repository (see Data availability section).*
2. *To enable targeted look-ups of eQTLs or pQTLs for specific genes, or look-ups of association for specific variants, the molecular QTL data are being integrated into the Musculoskeletal Knowledge Portal.*

In particular, for researchers interested in specific genes or variants, the search functions and visualisations of the Musculoskeletal Knowledge Portal will offer substantially enhanced functionality compared to making selected molecular QTL data available as supplementary tables. The portal will also enable researchers to interrogate the molecular QTL data for specific genes or proteins alongside genetic associations from GWAS, which are also integrated into the portal.

*As per the reviewer's suggestion, we have also explicitly considered whether variants that are significant pQTL also exhibit evidence of eQTL effects, and i****ncluded the results into the Supplementary Notes*** *(section "Identification of cis-eQTLs and cis-pQTLs in osteoarthritis tissues"):*
In low-grade cartilage, we detected 2871 pQTLs, of which 2866 are also present in the low-grade cartilage eQTL analysis. Of these variants, 2577 (90%) have the same direction of effect on gene

expression and protein levels, and 1295 (50%) were also significant eQTLs in low-grade cartilage at 5% FDR.
In high-grade cartilage, we detected 1971 pQTLs, of which 1969 were also present in the high-grade cartilage eQTL analysis. Of these variants, 1786 (91%) have the same direction of effect on gene expression and protein levels, and 1133 (63%) were also significant eQTLs in high-grade cartilage.

*We also display these results visually in **Supplementary Fig. 3d**.*

*Moreover, we have carried out a pathway enrichment analysis of genes with differential eQTL effects between high-grade and low-grade cartilage. We have not observed any significant results and have **added this analysis to the Supplementary Notes** (section "Differential eQTLs between low-grade and high-grade cartilage").*
We also acknowledge that this analysis had some limitations (it did not account for several factors that influence the power to detect eQTLs, including the expression levels of genes, the number of SNPs within the gene region, their LD pattern and minor allele frequency). Unfortunately, due to the small number of genes, the power to detect any significant associations is limited, so we have left more in-depth enrichment analyses of genes with differential eQTLs for future work based on larger sample sizes.

*We have also interrogated the STRING database for interactions between the 38 proteins with pQTLs detected in this study. The database includes interactions that have been experimentally determined or are listed in curated databases, along with other types of "interactions" (e.g. known gene fusions or co-occurence in literature determined by textmining). The resulting connections between proteins are shown in **Supplementary Fig. 4**.*
While the STRING analysis suggested the network has significantly more connections than expected ($p=3.07x10\text{-}8$), we again acknowledge that this analysis did not account for factors such as the abundance level of the proteins that make it more likely to detect both pQTL effects and interactions between proteins.
*We have also **added this analysis to the Supplementary Notes** (section "Identification of cis-eQTLs and cis-pQTLs in osteoarthritis tissues").*

4) Differential eQTLs were identified between low- and high grade cartilage, but not between cartilage and synovium. A major asset of this study is the inclusion of several disease-relevant tissues (synovium and cartilage), but this data is not really presented in the paper. It is remarkable that almost a fifth of the eQTLs in OA specifically occur in the synovium, but not in the cartilage. It would be of high interest to know which ones they are, what their function is and whether these could be therapeutic targets for OA.

The reviewer is right that for about 17% of genes, we detected a significant eQTL variant in synovium but not in cartilage. However, we expect that many of these associations may be present in other tissues and be detected in the future with larger sample sizes.
*Supporting this, we found generally high correlation of effect size estimates across all variant-gene pairs with significant association in at least one tissue (**Supplementary Fig. 2c**; Spearman correlation rho=0.86 for normalised effect sizes in synovium and low-grade cartilage, and rho=0.88 for synovium and high-grade cartilage).*

For low-grade and high-grade cartilage, the differential eQTL analysis can help identify genes with divergent regulatory effects that depend on the cartilage degeneration.
The conclusions from a differential eQTL analysis comparing cartilage and synovium would be a bit more complicated: reasons for different effects could be different roles genes have in these tissues, so a differential eQTL effect would not necessarily indicate potential involvement of a gene in osteoarthritis, or its value as a therapeutic target.

More broadly, work by the Gene-Tissue Expression Consortium (GTEx) found that the number of genes with significant eQTL effects ("eGenes") strongly increased with the number of samples per tissue (Science 2020, 10.1126/science.aaz1776):

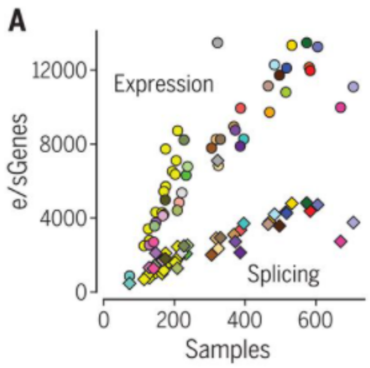

GTEx Fig 2. (A) The number of genes with a cis-eQTL (eGenes) or cis-sQTL (sGenes) per tissue, as a function of sample size.

Therefore, we expect that larger-scale studies of osteoarthritis disease tissue will help map out the tissue-specificity of regulatory effects.

Meanwhile, all molecular QTLs from our study will be made available to the broader community upon publication, both as large text files for computational approaches, and with visualisations and search function for targeted look-ups through the Musculoskeletal Knowledge Portal.

5) The **differential eQTLs** between high- and low grade cartilage are of interest, but also here the reader is a bit left alone with the results. Which pathways were enriched in low-grade cartilage, which in high-grade cartilage? The relationship between high and low grade cartilage should be discussed to help the reader to interpret the results. Is this considered as two different end-stage degrees of the disease or is low grade cartilage considered as precursor of high grade cartilage?

We thank the reviewer for the suggestion and have clarified the text and included additional information as follows.

Main text:
We now clarify in the first paragraph of the results section (underlined text added): "The availability of paired low-grade and high-grade cartilage samples enables the comparison between these two disease states in affected primary tissue, with high-grade cartilage showing more advanced cartilage degradation."

For each of "regulation of gene expression", "nervous system development", "response to stress", "immune response", "cell adhesion" and "catabolic processes", we clarified which genes have an effect present in high-grade cartilage and absent in low-grade cartilage, or vice versa.
We now summarise in the Results section: "For most of these processes, some genes with differential eQTLs show gain of genetic regulatory associations in high-grade cartilage, while others show loss of such associations compared to low-grade cartilage, suggesting a broader rewiring of regulatory processes."

***Additional information in Supplementary Notes** (section "Differential eQTLs between low-grade and high-grade cartilage"):*
Among 32 genes with differential eQTLs, 20 genes showed strong evidence for specific regulatory effects present in high-grade, but not low-grade cartilage; the remaining 12 genes showed strong evidence for specific regulatory effects present in low-grade, but not high-grade cartilage. We tested whether the two groups differed in genes annotated to the above processes. We did not find any significant associations (Fisher's test p>0.06), which could be due to a broader rewiring of regulatory processes (with some losses and some gains of genetic regulatory effects), and/or due to the small numbers of genes in the enrichment analysis.

We also tested whether genes with differential eQTL were enriched in any GeneOntology annotations with 20-200 genes using GoSeq, compared to all 1569 gene with an eQTL in any cartilage tissue. We separately analysed all 32 genes, as well as 20 respectively 12 genes based on tissue with observed differential eQTL effect, restricting the analysis to genes with unique Ensembl gene ID, gene symbol and Entrez gene ID. Again, we did not detect any significant associations (FDR>5%).

We note that this approach is simplified due to not accounting for several factors that influence the power to detect eQTLs, including the expression levels of genes, the number of SNPs within the gene region, their LD pattern and minor allele frequency. Given the limited power to detect associations based on a small number of genes, we have left more in-depth enrichment analyses of genes with differential eQTLs for future work based on larger sample sizes.

6) For the changes in gene/protein expression part of the study, maybe a heatmap showing the most differentially expressed genes/proteins could be shown as Figure. It would be interesting to learn more on the expected effect of the differential genes and pathways (e.g. lysosome, cytokine-cytokine receptor interactions). Furthermore, the effect of changed expression of some of the less known proteins found to be different between the two stages (e.g. the aldehyde dehydrogenases) could be elucidated with functional experiments, but this is probably out of the scope of the study.

*We thank the reviewer for the suggestion and have now included heatmaps of the most differentially expressed genes/proteins as **Supplementary Fig. 8**.*

We agree that it would be very interesting to elucidate the effect of changed expression of some of the less known genes and proteins via functional experiments, but these are outside the scope of the current study.

Caroline Ospelt

Reviewer #1 (Remarks to the Author):

All of my concerns have been addressed.

Reviewer #3 (Remarks to the Author):

All my comments have been addressed adequately. Thanks a lot and congratulations to the authors for their interesting analysis.
Caroline Ospelt
